# Psychological and physiological health outcomes of virtual reality-based mindfulness interventions: A systematic review and evidence mapping of empirical studies

**DOI:** 10.1177/20552076241272604

**Published:** 2024-10-25

**Authors:** Alissa Wieczorek, Florian Schrank, Karl-Heinz Renner, Matthias Wagner

**Affiliations:** 1 Faculty of Human Sciences, Institute of Sport Science, 558939 University of the Bundeswehr Munich, Neubiberg, Germany; 2 Faculty of Human Sciences, Institute for Psychology, 558939 University of the Bundeswehr Munich, Neubiberg, Germany

**Keywords:** Virtual reality, head-mounted display, mindfulness, health, meditation

## Abstract

**Objective:**

In the past two decades, mindfulness, rooted in Buddhist traditions, has gained considerable scientific interest. Virtual reality (VR) technology, in particular head-mounted displays, offers immersive experiences and is examined in this systematic review in terms of VR-based mindfulness interventions and their effects on psychological and physiological health outcomes.

**Methods:**

Using the Preferred Reporting Items for Systematic Review and Meta Analyses guidelines, a systematic search was conducted with the following search terms: [(mindful* OR “meditat*”) AND (“virtual reality” OR “VR”) AND (health OR physio* OR psycho* OR mental OR physical)]. Considering critiques of methodological quality in existing systematic reviews, this study adopts Boell and Cecez-Kecmanovic’s hermeneutic approach, critically evaluating research outcomes.

**Results:**

Psychological benefits include improved anxiety, mindfulness, emotions, disease patterns, affect, stress, (presleep) arousal, meditation and others. Physiological effects focus on neurobiological markers, heart rate/heart rate variability, pain, blood pressure, cortisol and galvanic skin resistance. Evidence mapping shows that more research has been conducted in the last 6 years, particularly by North American and South Korean authors, and points to gaps in study methodology. In addition, attention regulation is identified as a primary mindfulness mechanism in VR scenarios, often in nature-based virtual environments, with mainly single-session studies lasting 5 or 10 minutes.

**Discussion:**

Critical mapping reveals the need for additional studies to support and extend initial findings in this emerging research field. Methodologically, there is a call for more true-experimental studies to enhance rigor. From a content perspective, VR protocols are currently still strongly characterized by single-session interventions, which makes it especially difficult to make a dose–response statement regarding long-term effects.

**Conclusion:**

In summary, the studies provide important initial findings on psychological and physiological effects of VR-based mindfulness interventions on health. In addition, the need for more methodologically rigorous studies was emphasized, along with other methodological adjustments that must be carefully considered in the planning of future studies.

## Introduction

### Mindfulness and health

In the past 20 years, the scientific interest in mindfulness has surged covering different kinds of areas reaching from conceptualization to basic and applied science.^1,2^ Although the concept of mindfulness has been defined in different ways in the literature, Buddhist traditions are often seen as a substantial source and inspiration and has been described as a process of bringing a certain quality of attention to moment-by-moment experience.^3^ Bishop^4^ adopted an operational definition of mindfulness in order to specify testable theoretical predictions for the purpose of validation and refinement. This two-component model of mindfulness covers today’s general understanding of mindfulness, which can be described as a kind of nonelaborative, nonjudgmental, present-centered awareness in which each thought, feeling or sensation that arises in the attentional field is acknowledged and accepted as it is.^3,5–8^ In detail, the two components combine aspects of (a) self-regulation of attention and (b) orientation to experience by including the ability to sustain or switch the focus of attention and to inhibit secondary elaborative processing of thoughts feelings and sensations in the stream of consciousness. The first component also addresses the so-called “beginner’s mind” in order to widen the own experience and get into a state of direct observation, which is not filtered through own beliefs, assumptions, expectations or desires. The second component focuses on the aspect of dispositional openness, which describes a nonjudgmental attitude of curiosity and receptivity to new experiences. In adopting this stance of curiosity and acceptance less behavioral/cognitive strategies and improved affect tolerance are expected, allowing any thoughts, emotions and sensations to occur without further elaboration.^5^

Mindfulness has been introduced to the field of psychology, especially with respect to the clinical context, by commonly evaluated therapeutic approaches like the mindfulness-based stress reduction,^3^ the mindfulness-based cognitive therapy^8,9^ or the acceptance and commitment therapy.^10^ Each of these mindfulness-based interventions (MBIs) has originally been conceptualized for different target groups like patients in pain, with depression or social anxiety. Nevertheless, these approaches are constantly adapted for further populations, respectively focus on different contents like acceptance, mindfulness, emotion regulation or meditation techniques.^11^ These MBIs have been theoretically and empirically associated with psychological and physiological health outcomes showing the important contribution mindfulness can provide to one person's health. Concerning psychological health outcomes, beneficial effects of MBIs could be found for example in the improvement of well-being and behavioral regulation,^12^ positive affect^13,14^ as well as in reductions of anxiety and depressive symptoms,^15–18^ stress^19,20^ and burnout.^21^ Concerning physiological health outcomes, MBIs show varying positive bodily changes like reductions in heart and respiratory rates, hypertension symptoms,^22,23^ skin conductance^24^ and pain levels^25,26^ or an increase in heart rate variability (HRV).^27^

To theoretically frame these positive effects of MBIs on health, we apply Antonovsky's salutogenic model of health.^28^ This model views health as a continuum ranging from ease to dis-ease,^29^ reflecting a paradigm shift from pathogenesis to salutogenesis. This shift signifies a fundamental change in healthcare, emphasizing the promotion and maintenance of health and well-being rather than merely treating disease. This reorientation of healthcare toward a resource-orientated, salutogenic paradigm has been advocated by the Word Health Organization for several decades, as highlighted in the Ottawa Charter.^30^ With the mentioned continuum, Antonovsky^28^ wants to break away from the traditional medical dichotomy of sick/healthy in the pathogenic paradigm and views people from the perspective of heterostasis and entropy in the course of their lives as partly healthy and partly sick. Therefore, the salutogenesis approach stems from the idea of understanding health and disease not as alternative, dichotomous states, but as conceptual endpoints in a common continuum of health-ease (HE) and dis-ease (DE).

The primary objective in both life and research is to determine how individuals can progress toward the healthy end of the continuum, given the constant exposure to changes and events that may be considered as stressors. At this point, two key concepts of the salutogenesis model will be introduced. First, we will look at the generalized resistance resources (GRRs). This term was also shaped by Antonovsky^31^ and refers to the resources that facilitate the individual's abilities to cope effectively with stressors in avoiding disease. They can be of genetic, constitutional, psychosocial, cultural, spiritual and material nature and exist at the individual, group (family), subcultural and societal levels. GRRs are fundamental to developing a strong sense of coherence (SOC), the second key concept of the salutogenesis model we wanted to introduce. SOC reflects a person's view of life and capacity to respond to stressful situations, embodying a global orientation that perceives life as comprehensible, manageable and meaningful, guiding individuals to think, act and live with an inner trust that enables them to identify, benefit from, utilize and reuse available resources.^32^ The connection between GRRs and the SOC with its three elements comprehensibility, manageability and meaningfulness could be shown by a thematic analysis by Griffiths et al.,^33^ in which various general resistant resource themes such as coping strategies, challenges worth investing time and effort or solution focused outlook fit the SOC concept. Thus, GRRs act as a kind of cornerstone for the development of a strong SOC.

Incorporating mindfulness into this framework as a “way of being,” which emphasizes living in an open and highly engaged manner, facilitates responding rather than merely reacting to life’s challenges.^3^ Mindfulness may function as a GRR, e.g., as a kind of coping strategy, that strengthens the SOC. For instance, mindfulness can enhance the sense of manageability by promoting more adaptive responses to life's challenges and moment-to-moment awareness, which facilitates openness and understanding of experiences. In addition, mindfulness can foster a sense of purpose in life simply by allowing space to explore meaning. These are all aspects with which a SOC is associated, namely seeing the world as comprehensible, manageable and meaningful. In this theoretical framework, mindfulness can therefore help a person to move along the HEDE continuum towards the ease-pole.

Close to this is the concept of resilience which describes an individual's capacity for dealing with adversity, whether that adversity originates internally or externally.^34^ In this context, mindfulness is a crucial resource for building individual resilience in the face of adversity. Practicing mindfulness fosters traits such as emotional control, healthy coping mechanisms and a strong sense of self-worth, which are essential for navigating life challenges and environmental stressors. This practice can also help individuals recognize that the psychological distress caused by adverse events is merely a natural and temporary mental reaction. This awareness can enhance their ability to tolerate and cope positively with such challenges. Both the theoretical classification of mindfulness in the salutogenesis model and with regard to resilience, mindfulness plays a mediating role in improving mental and physical health.

### Mindfulness, health and virtual reality

As the possibilities for using different technologies are constantly evolving, virtual reality (VR) exhibits particularly favorable features respectively components that make the VR experience compelling, known as the “three Is” of VR^35^: interaction, immersion and imagination. First, an interaction with the VR environment is actively possible. Second, immersion is the system's capability to adequately stimulate all human perceptual channels (sensory, vestibular, proprioceptive, interoceptive) whose level depends on the extension of the perceptual domains involved (multisensory integration) and on the accuracy, resolution and reactivity in production of the stimuli.^36^ Therefore, VR is intentionally designed to give participants a heightened sense of presence, characterized by the “illusion of being immersed in the computer-generated world as if it were a place they are visiting.”^37^ This powerful experience of psychological presence describes a state, where users are immersed to the point where they momentarily disregard the physical world and perceive themselves as truly “in” the virtual environment.^38^ Third, the imagination aspect refers to the mind’s ability to create and perceive experiences, objects and environments that are not physically present, thereby convincing users to believe something even if their representation is unreal.^35^ In other words, this allows users to freely explore the simulated world with their imagination, enabling them to see, touch, move and experience things in new ways and from different perspectives.^39^ Imagination is defined as the extent to belief a user feels within a virtual environment, despite knowing he or she is physically situated in another environment^35,40^ in contrast to immersion defined by the degree a user associates being within a virtual environment.^41^ Moreover, it is important to mention, that immersion and interaction have a direct effect on a user's level of imagination, which is dependent on the VR's input devices, graphics and objectives as not all VR setups attempt to emphasize all three features. For example, full immersion is achieved with a head-mounted display (HMD), semi-immersion with large projection or LCD screens and non-immersion with standard desktop setups using keyboards and mice.^40–42^

Due to these technologies, interventions, especially for the mental health domain, are getting more accessible to the broader public.^43^ The benefits of different VR technologies (VR, augmented reality (AR), mixed reality (MR) and extended reality (XR)) for improving mindfulness practices and health-related outcomes in adults were demonstrated in two systematic reviews that addressed similar research questions or foci as intended in our review, published in 2021 and 2022. On the one hand, key findings from the systematic review of Zhang and colleagues^44^ indicate that both mindfulness interventions and the interactive experience of VR alone can alleviate a broad range of physical and psychological symptoms. However, the results do not show that a VR condition enhances the effectiveness of MBIs. Although, the MBI + VR condition retained more participants than the MBI alone, which might be an important aspect for improving treatment adherence and motivation to practice mindfulness. On the other hand, Arpaia and colleagues’^45^ narrative review aimed to verify scientific evidence that VR technology improves mindfulness practice and therapeutic effectiveness of mindfulness respectively. Special focus was set on mindfulness mechanisms like decentering and interoceptive awareness.^46^ From the literature review, it can be concluded that VR enhances relaxation and self-efficacy, reduces mind wandering and preserves attention resources. The authors also presented a design proposal for upcoming trends in VR-supported mindfulness with special focus on the integration of bio-/neurofeedback data. In addition to the two aforementioned papers, other researchers have also explored this topic through different reviews. However, they typically focus more specifically on particular settings, specific health outcomes or targeted populations. For this reason, we will not go into details of the individual reviews below, but instead provide an overview of the current range of research in this area. For example, Mitsea et al.^47^ showed in their review that mindfulness training supported by immersive technology can significantly improve a wide range of cognitive/socio-emotional meta-skills, emotional regulation and outcomes related to mental/physical health, academic performance and well-being in the special education setting. Moreover, a scoping review by O’Connor et al.^48^ on the management of chronic pain found preliminary evidence suggesting that VR may enhance aspects of mindfulness practice, such as inducing relaxation, centeredness or distraction. These effects could potentially alleviate pain and improve sleep and mobility for some individuals. Reviews that focus on a specific target group, such as that by Mitsea et al.^49^ show that mindfulness training supported by immersive technology significantly improves a wide range of cognitive and socio-emotional meta-skills in people with various mental disorders. In contrast, Failla et al.^50^ concentrated on a nonclinical population and were able to show that mindfulness interventions mediated by VR systems were able to induce a significant reduction in negative mood states combined with increased mindfulness skills. This is not a complete summary of current reviews, but merely offers an overview. Based on these findings, especially by Zhang et al.^44^ and Arpaia et al.,^45^ the purpose of this paper is to clarify the effectiveness of immersive VR-based mindfulness practice on psychological and physiological health outcomes through an updated and adapted systematic review. After almost 2 years, it can be assumed that sufficient new research has accumulated, especially in fast-moving areas such as (VR-based) technology, to justify updating the review and incorporating new findings as Zhang et al.^44^ included studies until 2020 and Arpaia et al.^45^ until January 2021. In addition to the valuable insights gained by both research groups, narrative reviews, such as the one by Arpaia et al.,^45^ always entail certain limitations. Those can be found for example in the nonprotocol-based search method or data extraction, which might lead to a subjective selection bias. Moreover, the inclusion of studies can be guided by the author’s intuition and research experience and is not clearly predefined by selection criteria.^51^ Although Arpaia et al.^45^ have counteracted with the formulation of eligibility criteria following Preferred Reporting Items for Systematic Review and Meta Analyses (PRISMA) recommendations, the narrative character is particularly evident in the derivation of design proposals for the integration of biofeedback or neurofeedback. Zhang et al.^44^ gave a comprehensive insight into this research topic by including all types of XR (xReality) technologies such as AR, VR and MR. In addition to various definitional approaches, Rauschnabel et al.^52^ specifically distinguish AR from VR. The clear distinguishing feature is that in AR, the physical environment is at least partially part of the experience, whereas in VR it is not. Furthermore, a nuanced differentiation is established between the continuums of AR and VR, delineated by local presence (AR), denoting the degree to which virtual entities are perceived as authentically present and telepresence (VR), reflecting the extent to which an individual perceives themselves to be situated within the virtual environment. Taking these technological differences into account, it becomes clear that a differentiated view of possible health effects is required and will be addressed in our systematic review.

Derived from this and from a content point of view, our interest lies solely on immersive VR technology, especially HMDs as they provide a deeply immersive experience by completely filling the user’s field of vision and enabling free movement within the virtual environment, distinguishing them from stationary VR rooms or cave systems. They offer portability, personalized use, potential cost-effectiveness and ongoing technological advancements in display quality, resolution, ergonomics and tracking systems, contributing to the creation of highly realistic and immersive VR experiences. Previous reviews define the concept of VR much more broadly^44^ and/or focus on specific aspects of mindfulness like decentering and interoceptive awareness^45^ as just outlined.

On a methodological level, this paper will address the growing criticism that systematic reviews receive. Those points of criticism include “a set of methodologies characterized by tight focus, exhaustive search, high rejection-to-inclusion ratio and an emphasis on technical rather than interpretive synthesis methods.”^53^ Other authors criticize that systematic reviews are often poorly conducted^54^ or not suitably written for policy decision making^55^ as well as they often fail to adequately capture the complexity of real-world phenomena and influential contextual factors.^56^ At the center of the criticism is the often-mechanistic approach of the process, which is accompanied by a lack of intellectual contribution. This means that the highly structured approach to searching for and obtaining original studies sometimes comes at the expense of intellectual analysis and interpretation of the studies. To pick up on these points, Boell and Cecez-Kecmanovic's^57^ hermeneutic approach for conducting literature reviews and literature research puts an additional focus on describing, classifying and critically assessing the totality of research results. Evidence mapping is employed to enhance the visualization of key findings. Therefore, this systematic review integrates an intellectual analysis and interpretation of the studies into the protocol-based PRISMA approach in order to conduct a methodological review of high quality identifying the effectiveness of those interventions.

## Systematic literature review methodology

### Eligibility criteria

In accordance with the PICOS approach [population (P), intervention (I), control/comparators (C), main outcome (O) and study design (S)] the following inclusion/exclusion criteria were used.^58^

*Inclusion criteria:*

IC1. Human participants of any gender and age
IC2. Immersive VR-based interventions that promote mindful content like awareness, commitment, acceptance, nonjudgmental attitude, (re-)focus or meditation
IC3. Passive-inactive control, alternative-active control following another psychological training or no control at all
IC4. Psychological/mental health outcomes (e.g., anxiety, pain, emotions, stress or mindfulness), physiological/physical health outcomes (i.e., cortisol, HRV, heart rate or lactate response)
IC5. Empirical studies of any design

*Exclusion criteria:*

EC1. Papers not written in neither English nor German
EC2. Papers published in the form of a review, meta-analysis, abstract (one or two pages) or posters
EC3. Papers dealing with technical configuration of VR-based mindfulness interventions

### Information sources and search strategy

We conducted the systematic review according to the PRISMA guidelines.^59^ To identify potentially relevant articles, a comprehensive search was conducted by the authors including a variety of study types covering randomized controlled studies as well as pilot and case studies. The study design intentionally encompassed more than just randomized controlled trials (RCTs) due to the early stage of research in this field. Nonrandomized trials, simple before–after comparisons or case–control designs hold significance for a systematic review, particularly in uncovering unexplored advantages and drawbacks and supplementing evidence derived from RCTs.^60^ Six databases, IEEE (Institute of Electrical and Electronics Engineers), ACM (Association for Computing), Scopus, PubMed, Base and EBSCO (PsycArticles, PsycInfo, PsyIndex, Medline), with a link to health psychology and human–computer interaction themes were screened from the earliest available evidence to September 22nd, 2022. Relevant search terms were combined with Boolean conjunctions (OR/AND) and applied as follows: [(mindful* OR meditat*) AND (“virtual reality” OR “VR”) AND (health OR physio* OR psycho* OR mental OR physical)]. Depending on the settings options in the database, the language and document/study type filters were preset.

### Article selection, data collection and extraction strategy

**Figure 1.**
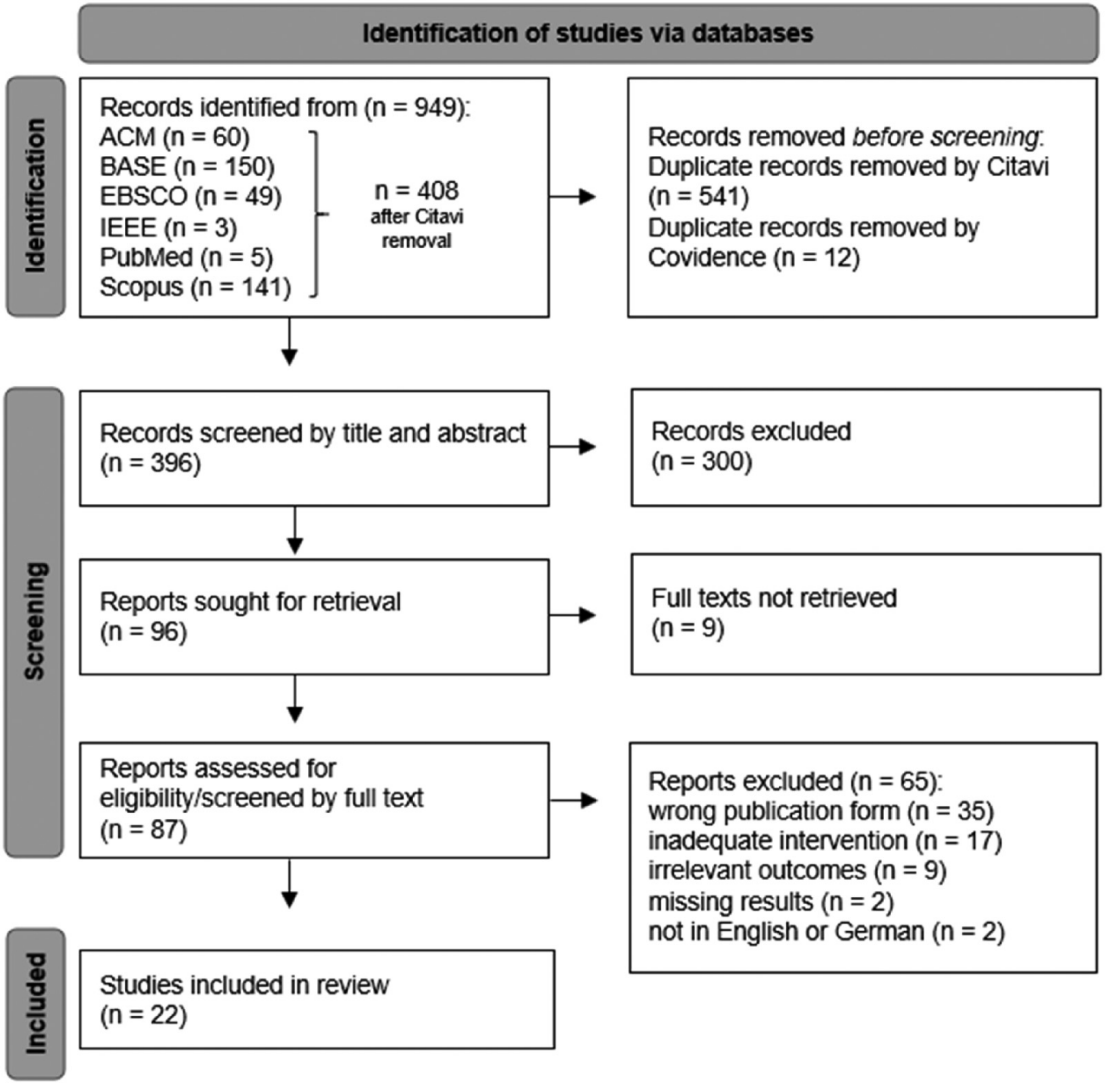
PRISMA flow chart (full texts not retrieved = no classic journal full text available, e.g., only as abstract format).

Search results (*n* = 949) were checked for duplicates (*n* = 541) with Citavi^®^^61^ and were then imported and processed in the Covidence systematic review management online system.^62^ During the selection process at Covidence additional duplicates (*n* = 12) were found and removed. The remaining articles were first identified for further review by screening the titles and abstracts. In case of insufficient information in title and abstract, a full article was required. Titles, abstracts and full texts were strictly screened according to the inclusion and exclusion criteria by the two reviewers (AW and FS). Any disagreements were always resolved through discussion between the two reviewers; a third reviewer would have been consulted if the discrepancy could no bet settled. The identification process in this systematic review is illustrated in Figure 1.

### Study risk of bias assessment

To assess risk of bias, different standardized tools were used in accordance with their study type and design respectively. For pre–post studies with no control group a checklist by the National Heart, Lung and Blood Institute^63^ was applied. This checklist is one of the few available tools in this study category. For case-series,^64^ quasi-experimental^65^ and RCT studies^66^ different Joanna Briggs Institute (JBI) checklists were chosen. The general choice of appraisal tools, especially for nonrandomized studies (NRSs), is complicated by the heterogeneity of this study category. It is often used as an umbrella term that encompasses a variety of designs such as experimental studies, quasi-experimental studies and traditional observational studies.^67^ Since there is no gold standard for NRSs, we have attempted to identify the most common and practical assessment tools for NRS types, preferably from one organization. Farrah and colleagues^67^ found that JBI checklists are one of the most commonly used in systematic reviews when multiple instruments are used according to different study designs. This prompted us to apply the three instruments mentioned. Any discrepancies that arose between the two raters (AW and FS) were identified and resolved through discussion. The full risk of bias assessment is provided in the Online Supplementary materials. Extracted data were used for the evidence mapping in order to describe, classify and critically assess the totality of research results.

## Results

### Literature search

The literature search resulted in 949 citations. After a first removal of duplicates by Citavi (*n* = 541), 408 documents were imported to Covidence^62^ and an additional 12 duplicates were found and removed before screening. In total, 22 studies were included in the systematic review. Reasons for exclusions are listed in the flowchart (Figure 1). Tables 1 and 2 provide a summary of the selected studies.

**Table 1.** Summary of included studies.

	Reference	Study type	Population			Intervention		
No.			EXP	CON	Target group, clinical condition/gender/age	VR/HMD technology	VR content	Length
1	Chandrasiri et al. (2020)	RCT	*n* = 16	*n* = 16	General population 16♀, 16♂ Age (years): *M* = 27.25 ± 6.04, range = 18–64	EXP: Oculus rift CON: –	A walk on the beach/360° landscape video of Australia's coast	EXP/CON: 1 × 20 minutes
2	Faraj et al. (2021)	Pre–post study with no control group	*n* = 15	–	MMT patients 11♀, 5♂ Age (years): 2 × 19–29, 5 × 30–49, 8 × 50+	Windows mixed reality headset	Different VR-guided meditations (a.o. KKC techniques)	12 weeks, 2 × 15 minutes/week
3	Feinberg et al. (2022)	Pre–post study with no control group	*n* = 15	–	Novice meditators Age (years): range = 18–34, median = 21	Oculus Quest	Zen VR interaction: progressive lessons to teach technical/philosophical foundations of meditation	4 weeks, 2 × 25 minutes/week
4	Frewen et al. (2020)	Case series	*n* = 10	–	Veterans 2♀, 8♂ PTSD self-reported symptom severity: *M* = 41.70 ± 6.55, range = 31–54	Dell Visor headset	Guided meditation VR	10 minutes introduction (choice of environment) + 5 minutes guided meditation (loving kindness vs. focused attention) + freely exploring VR environment using VR blink teleport functionality
5	Groninger et al. (2021)	RCT	*n* = 52	*n* = 36	Hospitalized participants with advanced heart failure 35♀, 53♂ Age (years): *M* = 56.1 ± 13.2	EXP: Oculus Go + over-the-ear headphones or device speakers CON: tablet + over-the-ear headphones or tablet speaker	EXP: forest of serenity (Holosphere VR^®^, Birmingham, UK) CON: meditation with instrumental background music + 2D imagery of a peaceful lakeside view	EXP/CON: 1 × 10 minutes
6	Hargett et al. (2022)	Pre–post study with no control group	*n* = 11	–	Hospitalized participants with preexisting opioid tolerance or opioid use disorder 6♀, 5♂	Oculus Go	Calm^®^ application (guided meditations incl. gratitude, impermanence, choice, grief etc.) 7	1 × 10 minutes
7	Hawes and Arya (2021)	RCT	*n*_meditation _= 26 2nd condition: primed vs. no-primed	*n*_game _= 30	(Non) students 30♀, 26♂ Age (years): range = 17–48	Oculus Quest	EXP: Calm^®^ application (VR-guided meditation) CON: Beat Saber Demo (VR game)	EXP: 1 × 5 minutes CON: 2 × 2 minutes 35 seconds
8	Kazzi et al. (2018)	Quasi-experimental study	*N* = 16 (two experiments)		Healthy participants 9♀, 7♂ Age (years): *M* = 34 ± 13, range: 20–68	Samsung Gear VR	EXP: VR 3D HMD active CON: VE 2D via phone passive CG: seated rest	EXP/active CON: 1 × 5 minutes guided relaxation/breathing passive CON: 1 × 5 minutes rest
9	Kim et al. (2022)	Pre–post study with no control group	*N* = 5	–	3♀, 2♂ Age (years): range = 21–35	Oculus Quest 2	Melody of the Mysterious Stones (mini games with the focus to create a flow experience)	Not specified
10	Kwon et al. (2020)	Pre–post study with no control group	*N* = 22	–	Typically developing children, no history of psychiatric illnesses Age (years): *M* = 11.59 ± 1.84, range = 9–16	Samsung Gear VR	VR scene day before exam (Exam1) → VR at-home meditation (Med1) → VR scene exam day (Exam2) → VR at-school meditation (Med2)	Exam1: approx. 3 minutes Med1: approx. 3 minutes Exam2: approx. 2 minutes Med2: approx. 4 minutes
11	Min et al. (2020)	Quasi-experimental study	*N* = 25		Paid subjects, 16♀, 9♂ Age (years): *M* = 23 ± 3.485, range = 19–35	Oculus CV1	“Drop the (heart) beat (rate)” → VR scenario + haptic device which gives simulated kinesthetic feedback of a beating heart VR (POS): calm, soothing content—haptic device with fluffy cover VR (NEG): disturbing, unnerving content—haptic device with wet cover	3 minutes 30 seconds. VR NEG or POS → 3 minutes rest → 3 minutes 30 seconds. VR NEG or POS (counter-balanced order)
12	Mistry et al. (2020)	Quasi-experimental study	*N* = 96 8 between groups conditions participants were randomized with counterbalancing (12 participants in each condition)		Undergraduate university students with different experience in meditation 54♀, 42♂ Age (years): range = 17–22	Dell Visor Windows mixed reality headset + headphones	Guided meditation VR	10 minutes introduction: choice of environment + music → 5 minutes VR/non-VR (randomized order + randomized content: FAM or LKM + randomized completion of meditation in non-VR group: eyes open or closed) → 5 minutes VR/non-VR
13	Nararro-Haro et al. (2016)	Case series	*N* = 1	–	32 years female with diagnosed borderline personality disorder and substance use disorder/polysubstance dependence/multiple hospitalizations due to drug overdose, suicide attempt, nonsuicidal self-injuries	Kaiser Electro-Optics goggles	1 month of standard DBT^®^ → 4 sessions of VR + DBT^®^: VR scenario with an illusion of floating down a 3-D-computer generated river + audio tracks (observing sounds, observing visuals, wise mind)	4 × 8–10 minutes (one session per week for 4 weeks)
14	Navarro-Haro et al. (2017)	Pre–post study with no control group	*N* = 44	–	Attendees of an international meeting with varying experience using computers and VR systems 28♀, 16♂ Age (years): *M* = 45.32 ± 13.20, range = 21–69	Oculus Rift DK2	VR + DBT^®^ mindfulness skills training: VR scenario with an illusion of floating down a 3-D-computer generated river + audio tracks (observing sounds, observing visuals, wise mind)	1 × 10 minutes (randomly assigned audio track)
15	Navarro-Haro et al. (2019)	RCT	MBI + VR: *n* = 19	MBI: *n* = 20	GAD diagnosed participants 30♀, 9♂ Age (years): *M* = 45.23 ± 11.23	Oculus Rift DK2	MBI: mindfulness -based program developed by García Campayo and Demarzo for both conditions together MBI + VR: VR + DBT^®^ mindfulness skills training	MBI: 7 × 90 minutes group sessions MBI + VR: 6 × 90 minutes group sessions + 6 × 15 minutes (10 minutes VR + DBT^®^/5 minutes measures)
16	Roo et al. (2017)	Quasi-experimental study	VR/SAR: *N* = 12 counterbalanced order of conditions		Participants interested in meditation and varying practical exercise 12♀ Age, years: 45 ± 11	Oculus Rift	VR: meditation with focus on breathing SAR: a sandbox is connected to physiological sensors to create a mindful interactive experience where the user can shape her own world that evolves according to breathing/heart rate	1st condition: VR → SAR 2nd condition: SAR → VR VR/SAR: 1 × 10 minutes
17	Semertzidis et al. (2019)	Pre–post study with no control group	*N* = 12	–	Nonclinical, healthy participants 3♀, 9♂ Age (years): *M* = 33 ± 11.86	VR headset (no specification)	Inter-dream system: neurofeedback modulating visuals (displayed in VR headset) + interactive bed with haptic feedback + sound	1 × 10 minutes
18	Seol et al. (2017)	Pre–post study with no control group	*N* = 5	–	Panic disorder patients	Oculus CV1	VR scenario: 1. neutral/peaceful state, 2. panic exposure/escape button, 3. “drop the beat”	2 sessions with 1–2 week break
19	Tarrant et al. (2018)	Quasi-experimental study	*n* = 14 Nonrandomized assignment	*n* = 12	GAD diagnosed participants 11♀, 3♂ (EXP) 9♀, 3♂ (CON) Age (years): *M* = 46.21 ± 10.77 (EXP), *M* = 48.17 ± 20.11 (CON)	Gear VR powered by Samsung Android S7 smartphone	EXP (VR): mindfulness in nature experience incl. 360° video photography + guided meditation CON: eyes-open resting period	EXP (VR): 1 × 5 minutes 41 seconds CON (rest): 1 × 5 minutes
20	Tarrant et al. (2022)	RCT	VR + neurofeedback: *n* = 50 Alternately assignment	Audio: *n* = 50	Frontline healthcare workers 43♀, 7♂ (EXP) 48♀, 2♂ (CON) Age (years): *M* = 42.16 ± 14.4 (EXP), *M* = 40.9 ± 13.9 (CON)	EXP: Oculus VR goggles (+Brainlink Lite EEG headband integrating high beta brainwaves as visual biofeedback) CON: over the ear headphones + iPad	VR: VR Healium scenario “relaxation beach” = body-scan/relaxation mindfulness meditation Audio: audio track of “relaxation beach”	EXP/CON: 1 × 1 minute warm up + approx. 5 minutes intervention
21	Wren et al. (2021)	Pre–post study with no control group	*n* = 61	/	Children + young adults diagnosed with inflammatory bowel disease (ulcerative colitis or Chron's disease) 26♀, 35♂ Age (years): *M* = 15.6 ± 3.29, range = 10–25	Samsung Gear VR + Samsung Galaxy S8 smartphone	MBVR = mindfulness-based VR intervention “MediMindfulness-Transitions” → aim: cultivating focused-attention + present moment awareness (breathing/natural environment)	1 × 6 minutes
22	Zambotti et al. (2022)	Quasi-experimental study	Good sleepers (SLE): *n* = 34 Insomnia sufferers (INS): *n* = 18 Counterbalanced order of intervention/control session		Junior and senior high-school students with/without insomnia disorder according to DSM-5 12♀, 6♂ (INS) 20♀, 14♂ (SLE) Age (years): *M* = 18.4 ± 0.7 (INS), *M* = 18.4 ± 0.8 (SLE)	Oculus Rift	Nature-based VR-guided meditation + paced breathing meditation (Nature Treks VR images of Greener Games)	EXP: 1 × 20 minutes VR meditation scenario CON: 1 × 20 minutes quiet activity of choice (e.g., watching TV, reading)

*Note.* VR: virtual reality; HMD: head-mounted display; RCT: randomized controlled trial; PTSD: posttraumatic stress disorder; MBI: mindfulness-based intervention; MMT: methadone maintenance treatment; KKC: Kids Kicking Cancer; FAM: focused attention meditation; LKM: loving kindness meditation; SAR: Spatial Augmented Reality; DSM: Diagnostic and Statistical Manual of Mental Disorders; PCG: postcentral gyrus.

**Table 2.** Measures and results of included studies.

Number	Reference	Outcomes	
		Measurement	Variables
1	Chandrasiri et al. (2020)	Toronto Mindfulness Scale (TMS)	Total mindfulness: *p* < .05 ↑ (within-group, VR/non-VR); *p* = 1.95 (between-group, VR vs. non-VR) Decentering: *p* < .05 ↑ (within-group, VR/non-VR); *p* < .05 (between-group, VR > non-VR) Curiosity: *p* = .102 (within-group, VR); *p* = .152 (within-group, non-VR); *p* = .932 (between-group, VR vs. non-VR)
2	Faraj et al. (2021)	Visual analogue scales (VAS) Saliva samples MRI scanning	Pain: *p* = .042, *η*^2^ = 0.265 (session × week rm-ANOVA, main effect of session) opioid craving/anxiety/depression: *p* < .005 (session × week rm-ANOVA, main effect of session) craving: *p* = .022 week 1–6 ↑/*p* < .05 week 7–9 ↓ (session × week rm-ANOVA, main effect of week) anger: *p* > .05, n.s. (no main or interaction effect) Cortisol: *p* < .001, *η*^2^ = 0.62 ↓ (session × week rm-ANOVA, main effect of session) c-reactive protein (CRP): *p* > .05 (no main or interaction effect) Pain neuromatrix activation Positive activation at baseline (from zero) in left/right PCG (postcentral gyrus): *p* < .001/*p* = .0123 Left PCG pain-related activity: *p* = 0.23 ↓ (baseline vs. post) Higher self-reported pain functional interference at baseline associated with higher pain-related activation in left PCG at baseline: *p* = .03, *r*(15) = .56 Higher baseline presession cortisol associated with higher pain-related activation in left PCG at baseline: *p* = .02, *r*(15) = .591 Higher baseline pre-/postsession cortisol levels associated with higher pain-related activation in right/left anterior insula (AI) at post-intervention: *p* < .05, *r* = .56 to .70 Higher baseline presession CRP associated with higher pain-related activation left PCG at baseline: *p* = .037, *r*(14) = .557 Higher baseline postsession CRP associated with higher pain-related activation in the right PCG and left AI: *p* = .021, *r*(12) = .652; *p* = .025, *r*(12) = .640Pain neuromatrix functional connectivity Positive/negative functional connectivity between left/right PCG and other pain neuromatrix regions like dorsal posterior insula or cerebellum: *p* < .001 (baseline) Changes in functional connectivity between left/right PCG and other pain neuromatrix regions like left caudate or left inferior parietal lobe: *p* < .001 (pre vs. post) Higher baseline pain severity associated with higher functional connectivity: *p* < .001 Higher baseline pain functional interference associated with higher functional connectivity: n.s. Higher baseline pain severity associated with higher functional connectivity at the postintervention time point: *p* < .001 Higher baseline functional interference associated with higher functional connectivity at the postintervention time point: *p* < .001 Higher baseline pain severity associated with changes in functional connectivity from pre- to postintervention: *p* < .001
3	Feinberg et al. (2022)	Mindfulness Awareness Attention Scale (MAAS) Cognitive and Affective Mindfulness Scale-Revised (CAMS-R) Perceived Stress Scale (PSS) Meditation questionnaire (10-point Likert scale)	MAAS scores: *p* = .011, Cohen's *d* = .96 ↑ (pre vs. post) CAMS-R scores: *p* < .01, Cohen's *d* = .89 ↑ (pre vs. post) PSS-scores: *p* = .059, Cohen's *d* = .55 ↓ Ability to meditate during the lesson: *p* < .001, Cohen's *d* = 1.43 ↑ (1st vs. 8th lesson) Overall ability to meditate: *p* < .0001, Cohen's *d* = 2.03 ↑ (1st vs. 8th lesson) Confidence in meditating on their own: *p* < .001, Cohen's *d* = 1.76 ↑ (1st vs. 8th lesson)
4	Frewen et al. (2020)	Modified Differential Emotional Scale (mDES) Buddhist Affective States Scale (BASS) Meditative Experience Questionnaire (MEQ) Symptom Checklist 10-Revised (SCL-10R) Self-report of symptoms of PTSD (PCL-5)	Positive/negative affect ratings: high degree of positive affect, very little negative affect Buddhist affective state: high level of certain affective states Meditative experience: lower “normative” meditative experiences Psychological distress: very rarely experienced psychological symptoms PTSD symptoms: very rarely experienced psychological symptoms → postintervention data; results are only at descriptive level!
5	Groninger et al. (2021)	Self-reported pain scores (Likert scale 0–10) Functional Assessment of in Chronic Illness Therapy in Palliative Care 14 item scale (FACIT-Pal 14) National Comprehensive Cancer Network Distress Thermometer	Pain (primary outcome) *p* < .0001 ↓ (within-group, VR pre–post); *p* = .0001 ↓ (within-group, guided imagery pre–post) *p* < .0001/*p* = .0012 ↓ (rm-ANOVA mixed model, time effect, VR/guided imagery) *p* = .0153 (postintervention comparison, VR scores < guided imagery scores) *p* = .0011 (post–pre comparison, VR score decrease > guided imagery score decrease) *p* = .0002/*p* = .0966 ↓ (24 hours postintervention compared to baseline, VR/guided imagery) *p* < .0001/*p* = .0019 ↓ (intention-to-treat analysis, within-group, pre vs. post, VR/guided imagery) *p* < .0001/*p* = .0628 ↓ (intention-to-treat analysis, within-group, pre vs. post, incl. originally assigned groups, VR/guided imagery) Quality of life (secondary outcome) *Total FACIT-Pal 14 score: p* = .0255 ↑ (pre vs. post, VR); *p* = .0060 ↑ (pre vs. post; guided imagery) *Total FACIT-Pal 14 score*: *p* = .0863, n.s. (postintervention comparison, VR vs. guided imagery) *Total FACIT-Pal 14 score: p* = .05615, n.s. (post–pre comparison, VR vs. guided imagery) “*I have been short of breath”: p* = .0272 ↑ (pre vs. post, VR); *p* = .6889, n.s. (pre vs. post, guided imagery) “*I have been short of breath”: p* = .7066, n.s. (postintervention comparison, VR vs. guided imagery) “*I have been short of breath”: p* = .2095, n.s. (post–pre comparison, VR vs. guided imagery) “*I am content with the quality of life right now”: p* = .12342, n.s. (pre vs. post, VR); *p* = .0416 ↑ (pre vs. post; guided imagery) “*I am content with the quality of life right now”: p* = .5475, n.s. (postintervention comparison, VR vs. guided imagery) “*I am content with the quality of life right now”: p* = .6859, n.s. (post–pre comparison, VR vs. guided imagery) distress (secondary outcome) *Total distress score*: *p* = .0255 ↑ (pre vs. post, VR); *p* = .0060 ↑ (pre vs. post; guided imagery) *Total distress score*: *p* = .6314, n.s. (postintervention comparison, VR vs. guided imagery) *Total distress score*: *p* = .9943, n.s. (post–pre comparison, VR vs. guided imagery)
6	Hargett et al. (2022)	Self-reported pain scores (Likert scale 0–10)	Pain: *p* = .03 ↓ (pre vs. post)
7	Hawes and Arya (2021)	Shortened version of State and Anxiety Inventory (STAI) University of California Matrix Reasoning Task (UCMRT)	Anxiety *p* = .021 (between-subjects, game > meditation) *p* < .0001 (within-subjects, primed vs. no-primed) *p* = .824 n.s. (within-subjects, primed/no-primed × game/meditation) *p* = .0003 (VR game priming, posthoc); *p* = .000007 (VR meditation priming, posthoc) *p* = .41, n.s. (Δ prime vs. no-primed: VR meditation vs. VR gaming) Cognitive bandwidth *p* = .232 n.s. (between-subjects, game vs. meditation) *p* = .009 (within-subjects, primed vs. no-primed) *p* = .061 n.s. (within-subjects, primed/no-primed × game/meditation) *p* = .0007 (VR game priming, posthoc); *p* = .34 (VR meditation priming, posthoc) *p* = .03 (Δ prime vs. no-primed: VR meditation vs. VR gaming)
8	Kazzi et al. (2018)	Finger blood pressure (SphygomoCor XCEL, ATCor Medical) Electrocardiogram (ECG; Nexfin, Edwards Life Sciences)	Blood pressure *Systolic blood pressure (SBP, mmHg): p* = .0238; *diastolic blood pressure (DBP, mmHg): p* = .966 (rm-ANOVA; seated rest vs. 2D vs. VE) *SBP: p* = .767 (following handgrip: rest vs. VE); *p* = .172 (following serial events: rest vs. VE) *DBP*: *p* = .794 (following handgrip: rest vs. VE); *p* = .769 (following serial events: rest vs. VE) *Mean arterial pressure (MAP):* n.s. (following handgrip: rest vs. VE); n.s. (following serial events: rest vs. VE) *Pulse pressure (PP):* n.s. (following handgrip: rest vs. VE); n.s. (following serial events: rest vs. VE) Heart rate (HR) *p* = .092 (rm-ANOVA; seated rest vs. 2D vs. VE) *p* = .448 (following handgrip: rest vs. VE); *p* = .775 (following serial events: rest vs. VE)Heart rate variability (HRV) *Total power (10^3 ^ms^2^): p* = .364 (rm-ANOVA; seated rest vs. 2D vs. VE); n.s. (following handgrip: rest vs. VE); n.s. (following serial events: rest vs. VE) *LF power (nu): p* = .585 (rm-ANOVA; seated rest vs. 2D vs. VE); n.s. (following handgrip: rest vs. VE); *p* < .01 (following serial events: rest < VE) *HF power (nu): p* = .526 (rm-ANOVA; seated rest vs. 2D vs. VE); n.s. (following handgrip: rest vs. VE); *p* < .05 (following serial events: rest > VE) *LF/HF ratio: p* = .117 (rm-ANOVA; seated rest vs. 2D vs. VE); n.s. (following handgrip: rest vs. VE); *p* < .05 (following serial events: rest < VE)
9	Kim et al. (2022)	Positive and Negative Affect Schedule (PANAS)	Positive affect schedule: *M*_pre_ = 20.2, SD_pre_ = 7.96; *M*_post_ = 14.2, SD_post_ = 5.02 ↓ Negative affect schedule: *M*_pre_ = 12.4, SD_pre_ = 6.28; *M*_post_ = 4.8, SD_post_ = 3.19 ↓ → Results are only at descriptive level!
10	Kwon et al. (2020)	Physiological measures, BIOPAC MP 160 system VAS anxiety, 0–10 scale Clinical scales: Korean Test Anxiety Inventors (K-TAI), Children's Depression Inventory (CDI), Korean State-Trait Anxiety Inventory (STAI-C)	Physiological measures (heart rate = HR; heart rate variability = HRV) *Heart rate* (see VAS anxiety results) *Root mean square of the successive differences (RMSSD, HRV): p* = .036 (mixed ANOVA; main time effect) *Average of NN (AVNN, HRV): p* = .05 (mixed ANOVA; main time effect); *p* < .05 (posthoc analysis, Session A vs. Session B) *Standard deviation of NN (SDNN, HRV): p* = .002 (mixed ANOVA; main time effect); *p* < .05 (posthoc analysis; Exam1—Med1/Exam2—Med2) *Proportion of NN50 (pNN50, HRV): p* = .199, n.s. (mixed ANOVA) VAS anxiety *p* = .0012 ↑ (rm-ANOVA posthoc; after Exam1); *p* = .001 ↓ (rm-ANOVA posthoc; after Med1); *p* = .006 (rm-ANOVA posthoc; after Exam2); *p* = .015 (rm-ANOVA posthoc, after Med2) VAS anxiety changes* ↔ average heart rate (AVHR): *r*(19) = .62, *p* = .003 (Session A); n.s. (Session B); *r*(19) = −.66, *p* = .001 (during Med1); *r*(19) = −.54, *p* = .011 (during Med2) Clinical scales *K-TAI* ↔ *CDI: r*(20) = .73, *p* < .001 (correlation) *VAS anxiety* ↔ *K-TAI: r*(20) = .53, *p* = .01 (correlation; pre-Med1); *r*(20) = .69, *p* < .001 (correlation; pre-Med2);*Session A: pre-Med1—baseline/Session B: pre-Med2—post-Med1
11	Min et al. (2020)	State and Anxiety Inventory (STAI) Physiological measures, ECG sensor (PSL-iECG2, PhysioLab)	(Mental) anxiety *POS: p* < .001, *d* = 1.404 ↓ (ANOVA, pre vs. post); *p* = .001, *d* = .231 (ANOVA, pre vs. post2/retrospective) *NEG: p* < .001, *d* = 2.635 ↑ (ANOVA, pre vs. post); *p* < .001, *d* = 1.089 (ANOVA, pre vs. post2/retrospective) Heart rate (HR) *POS: p* < .001 ↓ (ANOVA; pre vs. post) *NEG: p* = .0829, n.s. (ANOVA, pre vs. post)Blood pressure (BP) *POS: systolic BP with p* < *.001* ↓; *diastolic BP with p* = *.002* ↓ *(ANOVA, pre v. post)* *NEG: systolic P with p* = *.831, n.s.; diastolic BP with p* = *.564, n.s. (ANOVA, pre v. post)*Galvanic skin resistance (GSR) *POS: n.s. (ANOVA, pre v. post)* *NEG: p* = *.001* ↑ *(ANOVA, pre v. post)*
12	Mistry et al. (2020)	Modified Differential Emotional Scale positive/negative affect (mDES-PA/NA) Buddhist Affective States Scale (BASS) Meditative Experience Questionnaire (MEQ)	Positive affect *TYPE* (VR vs. non-VR): *p* < .001, *η*^2 ^= .472 (multivariate level) *TYPE* *×* *ORDER* (VR first/second vs. non-VR second/first): *p* < .001, *η*^2 ^= .338 (univariate level) → significant for all positive affective states besides “grateful, appreciative, thankful” (*p* = .173) + “love, closeness, trust” (*p* = .071) VR meditation first reported greater positive affect (follow-up within-group *t*-tests)negative affect: TYPE n.s.; TYPE *×* ORDER n.s. Buddhist affective states *TYPE: p* < .001, *η*^2 ^= .402 (multivariate level) *TYPE*: all BASS are significant (univariate level) besides “confusion, uncertainty, doubt” (*p* = .026, n.s.) *TYPE* *×* *ORDER*: *p* = .004, *η*^2 ^= .402 (multivariate level) *TYPE* *×* *ORDER*: significant for “absorption, immersion, engrossment” + “oneness, unity, connectedness” + “ecstasy, rapture, bliss” (univariate level) VR meditation first is associated with higher BASS (follow-up test within-group *t*-tests) Meditative experiences *TYPE*: *p* = .003, *η*^2 ^= .323 (multivariate level) → e.g., “less awareness of the presence of others in the room” *TYPE* *×* *ORDER: p* < .001, *η*^2 ^= .323 (multivariate level) → e.g., “feeling relaxed and calm”; n.s. (univariate level) VR meditation first is associated with e.g., greater “pleasant or happy thoughts or memories”; no differences for those who completed VR meditation second (follow-up within-group *t*-test) Group differences are restricted to response to the non-VR meditation, e.g., less “feeling relaxed an calm” by those participants receiving the VR meditation first (*p* = .001, follow-up between-group *t*-test)
13	Nararro-Haro et al. (2016)	Adapted DBT^®^ diary card to assess urges (0–5 scale) and emotions (0–100 scale) Adapted KIMS-short version	Urge to commit … *suicide/self-harm/quit therapy/use substances:* reduced after VR mindfulness exercise ↓ Emotions—*fear/anger/guilt/shame/disgust*: reduced after VR mindfulness exercise ↓ Emotions—*joy*: session 1 ↑, session 2 ↔, session 3 ↑, session 4 ↓ Mindfulness scores *Wised mind/observing sounds VR scenario*: increase in mindfulness scores ↑ *Observing visuals VR scenario:* slight decrease in mindfulness scores ↓→ Results are only at descriptive level!
14	Navarro-Haro et al. (2017)	Emotional State Scale (VAS) Mindfulness Awareness Attention Scale (MAAS)	Emotional state *Joy*: n.s., *d* = .06 (paired *t*-test, pre vs. post) *Sadness: p* < .01, *d* = .44 ↓ (paired *t*-test, pre vs. post) *Anger*: *p* < .05, *d* = .28 ↓ (paired *t*-test, pre vs. post) *Surprise*: n.s., *d* = .24 (paired *t*-test, pre vs. post) *Anxiety*: *p* < .01, *d* = .51 ↓ (paired *t*-test, pre vs. post *Relax: p* < .01, *d* = .68 ↑ (paired *t*-test, pre vs. post) *Vigor*: n.s., *d* = .02 (paired *t*-test, pre vs. post) State of mindfulness: *p* < .001, *d* = .75 ↑ (paired *t*-test, pre vs. post)
15	Navarro-Haro et al. (2019)	General Anxiety Disorder 7 Items (GAD-7) Hospital Anxiety and Depression Scale (HADS) Five Facets of Mindfulness Questionnaire (FFMQ) Difficulties of Emotion Regulation Scale (DERS) Multidimensional Assessment of Interoceptive Awareness (MAIA) Emotional state scale (7-point Likert scale)	GAD-7 (primary outcome): *p* < .001, *d* = −1.33 ↑ (within-group MBI + VR, pre vs. post); *p* < .001, *d* = −1.36 ↑ (within-group MBI, pre vs. post) HADS, anxiety and depression *Anxiety*: *p* < .001, *d* = −.96 ↓ (within-group MBI + VR, pre vs. post); *p* < .001, *d* = −1.27 ↓ (within-group MBI, pre vs. post) *Depression*: *p* = .027, *d* = −.54 ↓ (within-group MBI + VR, pre vs. post); *p* < .001, *d* = −1.33 ↓ (within-group MBI, pre vs. post) FFMQ, mindfulness aspects *Observing: p* = .244, n.s., *d* = .26 ↑ (within-group MBI + VR, pre vs. post); *p* = .133, n.s., *d* = .34 ↑ (within-group MBI, pre vs. post) *Describing*: *p* = .001, *d* = .85 ↑ (within-group MBI + VR, pre vs. post); *p* = .016, *d* = .73 ↑ (within-group MBI, pre vs. post) *Awareness*: *p* = .003, *d* = .66 ↑ (within-group MBI + VR, pre vs. post); *p* < .001, *d* = 1.07 ↑ (within-group MBI, pre vs. post) *Nonjudging: p* = .024, *d* = .55 ↑ (within-group MBI + VR, pre vs. post); *p* = .222, n.s. *d* = .65 ↑ (within-group MBI, pre vs. post) *Nonreactivity: p* = .152, n.s., *d* = .58 ↑ (within-group MBI + VR, pre vs. post); *p* = .383, n.s. *d* = .39 ↑ (within-group MBI, pre vs. post) DERS, emotion regulation *Inattention*: *p* = .172, n.s., *d* = −.30 ↓ (within-group MBI + VR, pre vs. post); *p* = .016, *d* = −.67 ↓ (within-group MBI, pre vs. post) *Confusion: p* = .014, *d* = −.59 ↓ (within-group MBI + VR, pre vs. post); *p* = .045, *d* = −.11 ↓ (within-group MBI, pre vs. post) *Nonacceptance: p* = .202, n.s., *d* = −.28 ↓ (within-group MBI + VR, pre vs. post); *p* = .011, *d* = −.76 ↓ (within-group MBI, pre vs. post) *Interference: p* < .001, *d* = −.84 ↓ (within-group MBI + VR, pre vs. post); *p* = .090, n.s., *d* = −.31 ↓ (within-group MBI, pre vs. post) *Impulse*: *p* = .001, *d* = −.89 ↓ (within-group MBI + VR, pre vs. post); *p* = .004, *d* = −.88 ↓ (within-group MBI, pre vs. post) Interoceptive awareness *Noticing*: *p* = .419, n.s., *d* = .29 ↑ (within-group MBI + VR, pre vs. post); *p* = .080, n.s., *d* = .37 ↑ (within-group MBI, pre vs. post) *Distraction*: *p* = .802, n.s., *d* = −.31 ↓ (within-group MBI + VR, pre vs. post); *p* = .892, n.s., *d* = −.02 ↓ (within-group MBI, pre vs. post) *Nonworrying*: *p* = .534, n.s., *d* = .21 ↑ (within-group MBI + VR, pre vs. post); *p* = .220, n.s., *d* = .28 ↑ (within-group MBI, pre vs. post) *Attention regulation*: *p* = .150, n.s., *d* = .29 ↑ (within-group MBI + VR, pre vs. post); *p* < .001, *d* = .72 ↑ (within-group MBI, pre vs. post) Emotional awareness: *p* = .534, n.s., *d* = .12 ↑ (within-group MBI + VR, pre vs. post); *p* = .582, n.s., *d* = −.17 ↑ (within-group MBI, pre vs. post) *Self-regulation: p* < .001, *d* = .98 ↑ (within-group MBI + VR, pre vs. post); *p* < .001, *d* = .89 ↑ (within-group MBI, pre vs. post) *Body-listening: p* = .016, *d* = .54 ↑ (within-group MBI + VR, pre vs. post); *p* < .001, *d* = .92 ↑ (within-group MBI, pre vs. post) Trusting: *p* = .005, *d* = .63 ↑ (within-group MBI + VR, pre vs. post); *p* = .002, *d* = .70 ↑ (within-group MBI, pre vs. post) Emotional state *Happiness* ↑*: p* = .083, n.s. (session 1, pre vs. post); *p* = .248, n.s. (session 2, pre vs. post); *p* = .160, n.s. (session 3, pre vs. post); *p* = .097, n.s. (session 4, pre vs. post); *p* = .361, n.s. (session 5, pre vs. post); *p* = .248, n.s. (session 6, pre vs. post) *Sadness* ↓*: p* = .018 (session 1, pre vs. post); *p* = .001 (session 2, pre vs. post); *p* = .046 (session 3, pre vs. post); *p* = .032 (session 4, pre vs. post); *p* = .046 (session 5, pre vs. post); *p* = .265, n.s. (session 6, pre vs. post) *Anger* ↓: *p* = .025 (session 1, pre vs. post); *p* = .091, n.s. (session 2, pre vs. post); *p* = .033 (session 3, pre vs. post); *p* = .164, n.s. (session 4, pre vs. post); *p* = .041 (session 5, pre vs. post); *p* = .212, n.s. (session 6, pre vs. post) *Surprise*↓/↑: *p* = .009 (session 1, pre vs. post); *p* = .086, n.s. (session 2, pre vs. post); *p* = .399, n.s. (session 3, pre vs. post); *p* = .285, n.s. (session 4, pre vs. post); *p* = .040 (session 5, pre vs. post); *p* = .714, n.s. (session 6, pre vs. post) *Anxiety* ↓: *p* = .002 (session 1, pre vs. post); *p* = .005 (session 2, pre vs. post); *p* = .067, n.s. (session 3, pre vs. post); *p* = .065, n.s. (session 4, pre vs. post); *p* = .001 (session 5, pre vs. post); *p* = .049 (session 6, pre vs. post) *Relaxation* ↑: *p* = .001 (session 1, pre vs. post); *p* = .001 (session 2, pre vs. post); *p* = .022 (session 3, pre vs. post); *p* = .003 (session 4, pre vs. post); *p* = .006 (session 5, pre vs. post); *p* = .015 (session 6, pre vs. post) *Vigor* ↓/↑: *p* = .166, n.s. (session 1, pre vs. post); *p* = .295, n.s. (session 2, pre vs. post); *p* = .763, n.s. (session 3, pre vs. post); *p* = .999, n.s. (session 4, pre vs. post); *p* = .033 (session 5, pre vs. post); *p* = .248, n.s. (session 6, pre vs. post)
16	Roo et al. (2017)	Toronto Mindfulness Scale (TMS)	TMS score: *p* = .13, n.s. (VR vs. SAR)
17	Semertzidis et al. (2019)	Pre-Sleep Arousal Scale (PSAS) Positive and Negative Affect Schedule Extended (PANAS-X) Muse^®^ EEG headset	PSAS, pre-sleep arousal *Somatic symptoms: p* = .25, n.s., *d* = .35 ↓ (paired *t*-test, pre vs. post) *Cognitive symptoms: p* = .01, *d* = .28 ↓ (paired *t*-test, pre vs. post) PANAS-X, emotion and affect *General negative emotion: p* = .008, *d* = .90 ↓ (*t*-test, pre vs. post) *General positive emotion*: *p* = .07, n.s., *d* = .30 ↑ (*t*-test, pre vs. post) *Basic negative affect: p* = .004, *d* = .90 ↓ (*t*-test, pre vs. post) *Basic positive affect: p* = .47, n.s., *d* = .15 ↑ (*t*-test, pre vs. post EEG data (frequency bandwidth most prevalent by measure of absolute power): *Delta (1–4 Hz): M* = .61, SD = .52 *Theta (4–8 Hz): M* = .10, SD = .35 *Alpha (7.5–13 Hz): M* = .29, SD = .16 *Beta (13–30 Hz): M* = .21, SD = .24 *Gamma (30–44 Hz): M* = .01, SD = .29
18	Seol et al. (2017)	Hamilton Anxiety Rating Scale (HAM-A) Beck Anxiety Inventory (BAI) Korean Version of the Montgomery-Asberg Depression Scale (K-MADRS) State and Anxiety Inventory (STAI)	HAM-A, anxiety: scores ↓/↑ (pre vs. post, 2 sessions) BAI, anxiety: scores ↓/↑ (pre vs. post, 2 sessions) K-MADRS, depression: scores ↓/↑ (pre vs. post, 2 sessions) STAI *State:* scores ↓/↑ (pre vs. post, 2 sessions) *Trait:* scores ↓/↑ (pre vs. post, 2 sessions)→ Results are only at descriptive level
19	Tarrant et al. (2018)	State and Anxiety Inventory (STAI, form Y/state portion) EEG eyes-closed recordings with 19 electrodes in the standard 10–20 international placement General Anxiety Disorder 7 Items (GAD-7)	STAI *Scores*: *p* < 0.001, *η*_p_^2 ^= .60 ↓ (2 × 3 ANOVA, group × time, main effect for time); *p* = .202, n.s., *η*_p_^2 ^= .07 (2 × 3 ANOVA, no group effect); *p* = .54, n.s., *η*_p_^2 ^= .02 (2 × 3 ANOVA, no interaction effect) *Correlation analyses: STAI vs. High-Beta (T3-2): r* = .456, *p* = .019 Mean power, cognitive state *Alpha*: *p* = .002, *η*_p_^2 ^= .23 ↑ (ANOVA, time effect); *p* = .039, *η*_p_^2 ^= .17 (ANOVA, group effect, VR meditation > quiet rest); no interaction effect; *p* = .058, n.s., *p* = .036, *p* = .033 (follow-up ANOVA; T1: VR meditation ≈ quiet rest, T2/T3: VR meditation > quiet rest); *p* = .998, n.s. (T1-2, after rest interval, VR intervention); *p* = .369, n.s. (T1-2, after rest interval, quiet rest); *p* = .302, n.s. (T2-3, after VR meditation); *p* = .149, n.s. (T2-3, after quiet rest) *Alpha1: p* = .004, *η*_p_^2 ^= .20 ↑ (ANOVA, time effect); no interaction effect *Alpha2*: *p* = .006, *η*_p_^2 ^= .004 ↑/↓ (ANOVA, time effect); no interaction effect *Beta: p* = .005, *η*_p_^2 ^= .20 ↑ (ANOVA, time effect); no interaction effect *Low beta: p* = .001, *η*_p_^2 ^= .26 ↑ (ANOVA, time effect); *p* = .032, *η*_p_^2 ^= .18 (ANOVA, group effect, EXP > CON); no interaction effect; *p* = .036, *p* = .040, *p* = .025 (follow-up ANOVA, T1/T2/T3: VR meditation > quiet rest); *p* = .582, n.s. (T1-2, after rest interval, VR meditation); *p* = .205, n.s. (T1-2, after rest interval, quiet rest); *p* = .021 (T2-3, after VR mediation); *p* = .137 (T2-3, after quiet rest) *High beta: p* = .085, n.s., *η*_p_^2 ^= .098 (ANOVA, no time effect); no interaction effect Power ratios, spectral power shifts from the perspective of relative power changes *Alpha/Sum: p* = .015, *η*_p_^2 ^= .16 (time effect); no group-differences or interaction effects *Alpha1/Sum: p* = *.025, η*_p_^2 ^= .14 (time effect); no group-difference effect; *p* = .031 ↑ (T2-3; after VR meditation/quiet rest) *Alpha2/Sum: p* = .093, n.s., *η*_p_^2 ^= .09 (no time effect), no group-difference effect *Alpha1/Alpha2: p* = .375, n.s., *η*_p_^2 ^= .04 (no time effect), no group-difference effect *Alpha/Beta: p* = .009*, η*_p_^2 ^= .18 (time effect); no group-differences or interaction effects *Beta/Sum: p* = .523, n.s., *η*_p_^2 ^= .03 (no time effect); no group-differences or interaction effects *High-Beta/Sum: p* = .009, *η*_p_^2 ^= .18 (time effect); no group-difference effect; *p* = .009 ↓ (T1-3; VR meditation > quiet rest) *Low-Beta/Sum: p* = .660, n.s., *η*_p_^2 ^= .02 (no time effect), no group-difference effect *High-Beta/Alpha: p* < .001, *η*_p_^2 ^= .28 (time effect); no group-difference effect; *p* = .002 ↓ (T2-3, VR meditation/quiet rest) *Low-Beta/Alpha: p* = .008, *η*_p_^2 ^= .18 (time effect); no group-difference effect; *p* = .028 ↓ (T2-3, VR meditation/quiet rest) *Low-Beta/High-Beta: p* < .001, *η*_p_^2 ^= .36 (time effect), no group-difference effect; *p* = .032, *η*_p_^2 ^= .13 (interaction effect); *p* = .053, n.s., *p* = .089, n.s., *p* = .040 (follow-up ANOVA, T1/T2: VR meditation ≈ quiet rest, T3: VR meditation ≠ quiet rest); *p* = .998, n.s. (T1-2, after rest interval, VR meditation); *p* = .578, n.s. (T1-2, after rest interval, quiet rest); *p* = .005 (T2-3, after VR meditation); *p* = .463, n.s. (T2-3, after quiet rest) sLORETA, specific anterior and posterior cortical regions of interest for changes in regional activity across spectral bands *ACC (anterior cingulate cortex):* *Alpha*: *p* = .649, n.s., *η*_p_^2 ^= .02 (no time effect); no group-difference effect *Beta: p* = .054, *η*_p_^2 ^= .11 (time effect); *p* = .038, *η*_p_^2 ^= .13 (time × group effect); *p* = .795, n.s. (T1-2, after rest interval, VR meditation); *p* = .171, n.s. (T1-2, after rest interval, quiet rest); *p* = .048 ↓ (T2-3, after VR meditation); *p* = .998, n.s. (T2-3, after quiet rest) *Theta: p* = .664, n.s., *η*_p_^2 ^= .02 (no time effect); no group-difference effect *PCC (posterior cingulate cortex)* *Alpha*: *p* = .017, *η*_p_^2 ^= .16 (time effect); *p* = .042 ↑ (T1-3, VR meditation/quiet rest); *p* = .045, *η*_p_^2 ^= .16 (VR meditation > quiet rest) Beta: *p* = .006, *η*_p_^2 ^= .19 (time effect); *p* = .015 ↑ (T1-3, VR meditation/quiet rest); *p* = .049, *η*_p_^2 ^= .15 (VR meditation > quiet rest) Theta: *p* = .106, n.s., *η*_p_^2 ^= .09 (no time effect) CSD (current source density): linear increase over time for both groups GAD-7, correlation analyses *GAD scores vs. Alpha/Sum: r* = −.47, *p* = .019 (T1); *r* = −.35, *p* = .080 (T2); *r* = −.43, *p* = .027 (T3) *GAD scores vs. Alpha1/Sum: r* = −.46, *p* = .018 (T1); *r* = −.35, *p* = .078 (T2); *r* = −.43, *p* = .029 (T3) *GAD scores vs. Alpha2/Sum: r* = −.12 to *r* = −.13, p > .20 (T1/T2/T3) *GAD scores vs. Low-Beta/High-Beta: r* = −.45, *p* = .023 (T1); *r* = −.43, *p* = .030 (T2); *r* = −.39, *p* = .048 (T3) *GAD scores vs. High-Beta/Sum: r* = .48, *p* = .013 (T1); *r* = .45, *p* = .022 (T2); *r* = .44, *p* = .025 (T3) *GAD scores vs. Low-Beta/Sum: r* = .10–.12, *p* > .50 (T1/T2/T3)
20	Tarrant et al. (2022)	Brunel Mood Scale	Anger: *p* = .268, n.s., *η*_p_^2 ^= .01 (no group × time interaction) *p* = .001, *η*_p_^2 ^= .29 (time effect, VR/meditation↓) *p* < .001, *η*_p_^2 ^= .29 (group effect, audio > VR) Tension *p* = .248, n.s., *η*_p_^2 ^= .01 (no group × time interaction) *p* < .001, *η*_p_^2 ^= .52 (main time effect, VR/meditation↓) *p* = .365, n.s., *η*_p_^2 ^= .01 (no group effect) Depression *p* = .299, n.s., *η*_p_^2 ^= .01 (group × time interaction) *p* < .001, *η*_p_^2 ^= .42 (time effect, VR/meditation↓) *p* = .254, n.s., *η*_p_^2 ^= .01 (no group effect) Vigor *p* < .001, *η*_p_^2 ^= .18 (group × time interaction, VR↓/audio↑) *p* = .190, n.s., *η*_p_^2 ^= .02 (no time effect) *p* < .001, *η*_p_^2 ^= .24 (group effect, audio < VR) Fatigue *p* = .002, *η*_p_^2 ^= .10 (group × time interaction, VR↓/audio↑) *p* = .076, n.s., *η*_p_^2 ^= .03 (no time effect) *p* = .001, *η*_p_^2 ^= .10 (group effect) Confusion *p* = .003, *η*_p_^2 ^= .08 (group × time interaction, VR↓/audio ↔) *p* = .038, *η*_p_^2 ^= .04 (time effect, VR↑/audio ↔) *p* < .001, *η*_p_^2 ^= .14 (group effect) Happiness *p* < .001, *η*_p_^2 ^= .18 (group × time interaction, VR↑/audio ↔) *p* < .001, *η*_p_^2 ^= .15 (main time effect, VR/meditation ↑) *p* = .408, n.s., *η*_p_^2 ^= .01 (no group effect) Calmness *p* < .001, *η*_p_^2 ^= .32 (group × time interaction, VR↑/audio↓) *p* < .001, *η*_p_^2 ^= .22 (main time effect, VR/meditation ↑) *p* = .164, n.s., *η*_p_^2 ^= .02 (no group effect)
21	Wren et al. (2021)	Anxiety VAS scale Pain VAS scale	Anxiety *Preliminary efficacy: p* = .001 ↓ (pre vs. post) *Exploratory analyses*:< 18 years (*n* = 42): *p* < .001 ↓ (pre vs. post)> 18 years (*n* = 19): *p* = .41 ↓ (pre vs. post)Diagnostic subgroups: *p* > .05, n.s. (Crohn's disease vs. ulcerative colitis following the intervention) Pain *Preliminary efficacy: p* < .001. ↓ (pre vs. post) *Exploratory analyses*:< 18 years (*n* = 42): *p* = .001 ↓ (pre vs. post)> 18 years (*n* = 19): *p* = .16, n.s. (pre vs. post)Diagnostic subgroups: *p* > .05, n.s. (Crohn's disease vs. ulcerative colitis following the intervention)
22	Zambotti et al. (2022)	Pre-Sleep Arousal Scale (PSAS) Daytime Insomnia Symptom Scale, VAS (DISS) Physiological measures, EEG + ECG via Compumedics Grael^®^ system Saliva samples Automatic sphygmomanometers	PSAS, cognitive arousal: no group (between, insomnia sufferers vs. good sleepers), condition (within, VR intervention vs. control) or group × condition effects DISS, cognitive alertness/positive and negative mood/sleepiness: no group (between, insomnia sufferers vs. good sleepers), condition (within, intervention vs. control) or group × condition effects Physiological measures *EEG data:* No differences between resting state and following 20 minutes intervention/control in cortical activity Alpha power (occipital): *p* < .001, *η*_p_^2 ^= .08 ↓ (time main effect, pre vs. 20 VR intervention) Sigma power (central/occipital): *p* = .005, *η*_p_^2 ^= .06/*p* = .002, *η*_p_^2 ^= .07↑ (time main effect, pre vs. 20 VR intervention) Beta power (frontal/central/occipital): *p* = .002, *η*_p_^2 ^= .07/*p* < .001, *η*_p_^2 ^= .09/*p* < .001, * η*_p_^2 ^= 16 ↑ (time main effect, pre vs. 20 VR intervention) Gamma power (occipital): *p* < .001, * η*_p_^2 ^= .12 ↑ (time main effect, pre vs. 20 VR intervention) No further group, time or group × time effects *Heart rate (HR):* *p* < .001 ↓ (pre vs. post, VR meditation) → as well as levels preceding and following quiet activities (condition × time interaction: *p* = .004, * η*_p_^2 ^= 0.19) No HR difference in baseline periods preceding the intervention and quiet activities No between-group differences *Heart rate variability (HRV):* HF power: *p* = 0.039, * η*_p_^2 ^= 0.10 (group main effect, insomnia sufferers < good sleepers); no condition/time effect; *p* < .001, * η*_p_^2 ^= 0.18 (baseline vs. minutes 0-20 of VR meditation) LF power: no group difference; *p* < .001, * η*_p_^2 ^= 0.57 ↑ (baseline vs. minutes 0–20 of VR intervention) VLF power: *p* < .001, * η*_p_^2 ^= 0.14 ↑ (baseline vs. minutes 4–6, 8–10 of VR intervention) Total power: no group difference 4. Saliva, cortisol: *p* < .001, *η*_p_^2 ^= 0.25 ↓ (condition main effect) 5. Blood pressure (BP): no significant effects for SBP/DBP

In the following, we offer a concise overview of the psychological and physiological effects from VR-based mindfulness interventions as identified in the studies included. These studies are then subject to a further and deeper analysis employing the hermeneutic approach proposed by Boell and Cecez-Kecmanovic,^57^ wherein we categorize and map them across distributional, methodological and content-related perspectives.

#### Psychological effects

The selected studies showed a broad spectrum of psychological effects of VR-based mindfulness interventions. These effects were observed among both healthy individuals and those in a prediseased state. In general, the following positive effects on psychological variables could be found: anxiety,^69–76^ mindfulness,^74,77–81^ various emotions or emotional states like anger, happiness or vigor,^70,74,79,80,82,83^ different disease patterns like depression or posttraumatic disorder,^70,72,74,75,83,84^ affect,^84–86^ stress and distress respectively,^78,84,87^ (presleep) arousal,^82,88^ meditation experience^78,84,86^ and others like quality of life or opioid craving.^70,87^ It's worth noting that among the 22 studies, four only assessed their findings concerning psychological outcome variables descriptively.^75,79,84,85^

Effect sizes overall, including psychological and physiological outcomes, are only represented in half of the included studies. Looking into psychological outcome categories seven studies reported their results with effect sizes.

Concerning anxiety, as the most addressed physiological outcome, large effect sizes in reference to Cohen's guidelines^89^ with *d* > |.96| are represented in the studies by Navarro-Haro et al.^74^ and Tarrant et al.^76^ respectively with *η*_p_^2 ^= .60. Furthermore, Min et al.^73^ reported small to medium effects with *d* = 2.31, while Tarrant et al.^76^ observed effect sizes ranging from *η*_p_^2 ^= .02 to *η*_p_^2 ^= .07. Large correlation effects between anxiety and depression questionnaires (*r* = .73) respectively between two anxiety scales (*r* > .53), a self-report scale and a visual-analog scale, are reported by Kwon et al.^72^ Mindfulness effects exhibit substantial magnitudes, ranging from *d* = .96 to *d* = 1.07, as indicated by research conducted by Feinberg et al.^78^ and Navarro-Haro et al.^74,80^ In addition, small (*d* = |.02|) and medium to large (*d* = .26) effects are present in Navarro-Haro et al.^74^ Emotions and emotional states show a wide range of effect sizes, reaching from small (*d* = .02/*η*_p_^2 ^= .01) to large (*d* = |.89|/*η*_p_^2 ^= .52), depending on the ones examined by Navarro-Haro et al.,^74,80^ Semertzidis et al.^82^ or Tarrant et al.^83^ Concerning disease patterns, Navarro-Haro et al.^74^ are the only ones reporting an effect size with medium to large effect (*d* = −.54) for depression. Large effect sizes in affect variables are present in the study by Mistry et al.^86^ (*η*^2 ^= .338–.472) and Semertzidis et al.^82^ (*d* = .90). However, the latter study also records small effects in the area of affect variables with *d* = .15. Positive effects in the area of stress and distress are solely reported with a medium to large effect (*d* = .55) in Feinberg et al.^78^ Moreover, small to medium effects could be detected in presleep arousal by Semertzidis et al.^82^ Concerning meditation experiences, large effects (*d* > 1.43/*η*^2 ^= .323) could be found in the studies by Feinberg et al.^78^ and Mistry et al.^86^ In the studies from our category “others,” no effect sizes were provided by Faraj et al.^70^ or Groninger et al.^87^

#### Physiological effects

The range of positive effects of VR-based mindfulness interventions on physiological variables is less diverse, the following outcome variables were identified: neurobiological markers including electroencephalogram (EEG) data of different brain waves, pain neuromatrix activation or cognitive bandwidth,^70,71,76,82,88^ heart rate and HRV,^72,73,88,90^ pain,^69,70,87,91^ blood pressure,^73,88,90^ cortisol respectively saliva samples^70,88^ and galvanic skin resistance.^73^

Looking at the results within these categories, three studies supported them by reporting effect sizes, which are also categorized in reference to Cohen's guidelines.^89^ For example, neurobiological markers, primarily including EEG data, reached effect sizes from small (*η*_p_^2 ^= .004) to large (*η*_p_^2 ^= .57) as provided by the studies from Tarrant et al.^83^ and Zambotti et al.^88^ In addition, Faraj et al.^70^ showed different pain neuromatrix correlations, based on magnetic resonance imaging scans, with large effects (*r* > .56). The same study reported large effects regarding pain with *η*^2 ^= .265. Large effects in heart rate (*η*_p_^2 ^= .19) and medium to large effects in HRV (*η*_p_^2 ^> .10) were also detected in Zambotti et al.^88^ Positive effects on cortisol, respectively saliva samples, were shown by Faraj et al.^70^ and Zambotti et al.^88^ with a large effect sizes of *η*^2 ^= .265 and *η*_p_^2 ^= .25. Variables like blood pressure and galvanic skin resistance were presented without information on effect sizes.

### Study risk of bias assessment

**Figure 2.**
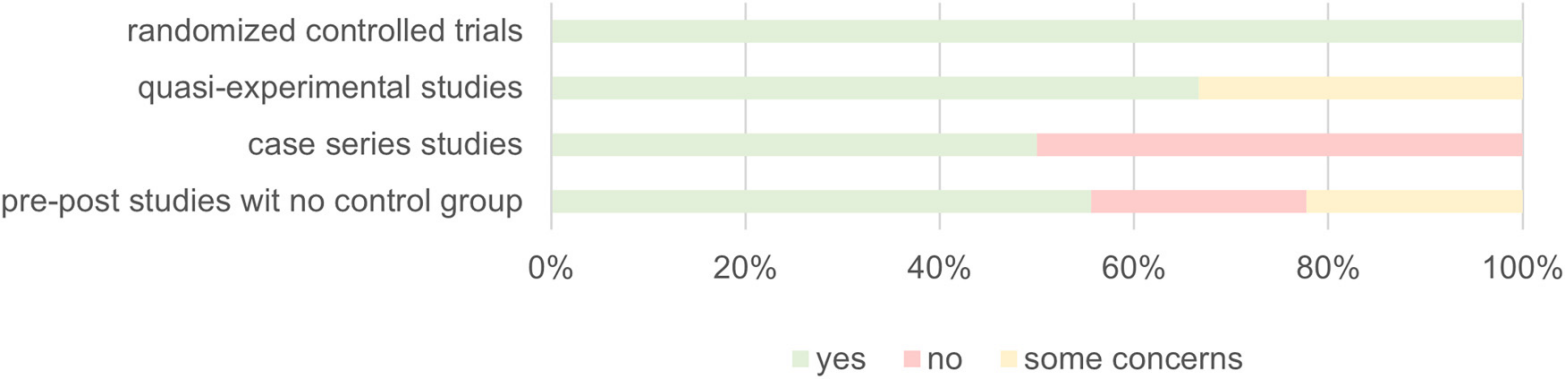
Summary of risk of bias assessment.

Figure 2 summarizes the results of the risk of bias assessment of methodological study quality using four standardized instruments. For this purpose, the respective ratings of the studies (see Online Supplementary materials) were used for an overall assessment and converted into the categories “yes,” “no” and “some concerns.” Pre–post studies with no control groups (*n* = 9) mostly lack in categories concerning sample size, blinding of outcome assessors and multiple outcome measures. Therefore, sample size was sometimes not sufficiently large enough to provide deep confidence as a priori analyses were largely nonexistent or the information whether outcome assessors were blinded was not given. In addition, outcome measures were mostly only recorded before and immediately after the intervention and no further or multiple measurements and especially no follow-up measurement to determine the stability of the effects was carried out. Nevertheless, 50% of the studies were rated with a good overall quality, 30% with fair and 20% with poor overall quality. The remaining studies were assessed through different JBI checklists. One of the case series (*n* = 2) studies was rated with poor quality due to a few unclear and not reported aspects like participants inclusion criteria, demographics of participants or appropriate statistical analyses. Reaching higher methodological study types, more than 60% of the quasi-experimental studies (*n* = 6) and 100% of the RCTs (*n* = 5) were rated with good overall quality even though some RCTs lacked in clearly reporting facts like allocation of groups or the blinding process. Some quasi-experimental studies revealed gaps in implementing a control group, multiple measurements and a follow-up in their study design.

### Critical assessment of results

In the subsequent section, a critical assessment of the studies and their outcomes is provided based on evidence mapping with different analysis aspects. The distributional analysis includes an examination of the diverse publication platforms featuring articles on VR-based mindfulness interventions and their potential health implications. Furthermore, an exploration of the temporal and geographical trends in the publication of studies within this domain is undertaken. The methodological analysis aims to recognize the principal research methodologies used in our studies on VR-based mindfulness interventions and their psychological and physiological effects. This involves a comprehensive examination of applied sampling methodologies and study designs including aspects like randomization techniques and the constitution of control groups. Concurrently, the content mapping seeks to identify psychological and physiological variables considered in these studies, especially those with a more robust evidential foundation concerning the research query. This analysis also attempts to evaluate previous VR protocols in terms of their intensity, duration and the integration of mindfulness elements within the VR scenarios.

In summary, this section encompasses a comprehensive analysis and classification of studies, covering their distribution across various publication channels, temporal and geographical trends, methodological approaches and content perspectives, thereby contributing to a holistic understanding of VR-based mindfulness interventions and their associated effects on health outcomes.

#### Distributional mapping of space, time and publication

**Figure 3.**
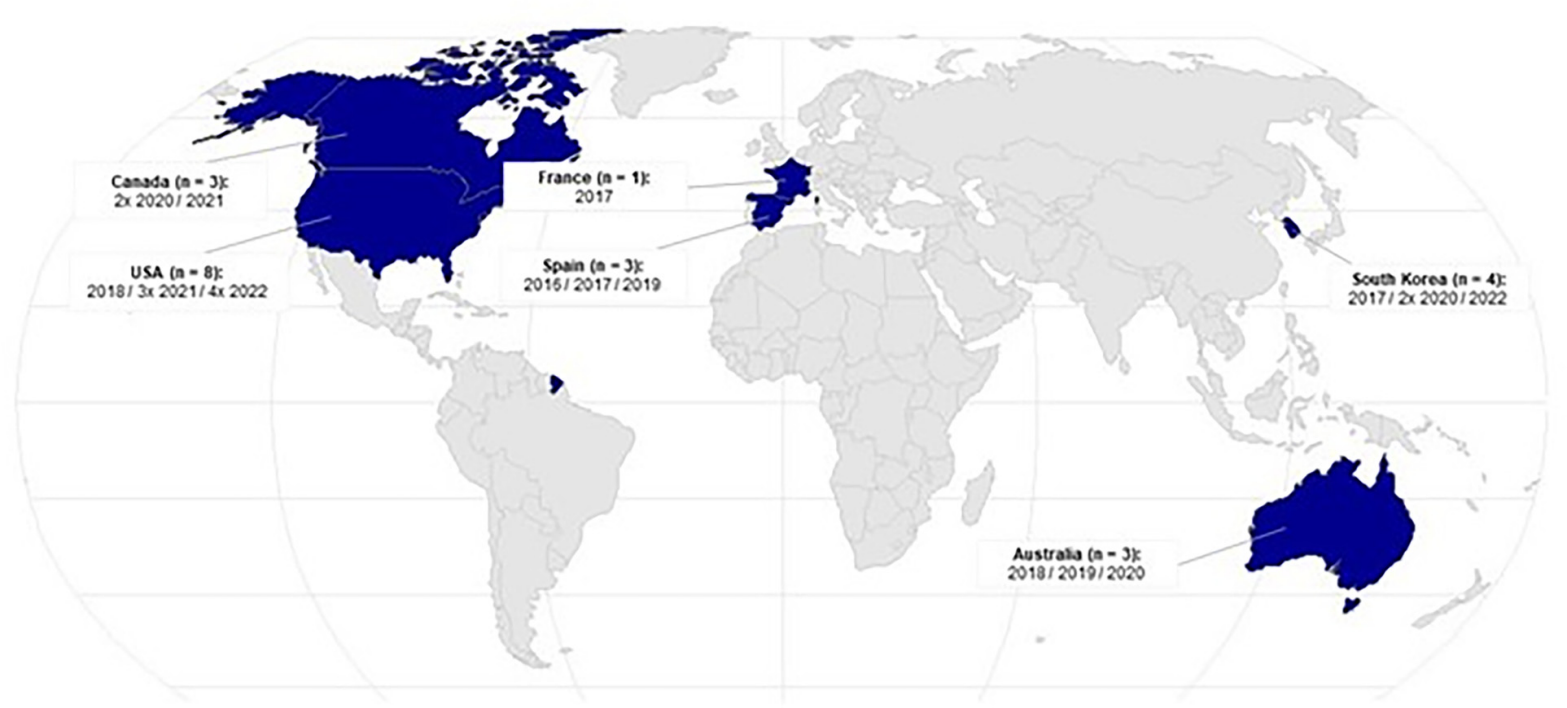
Geographical distribution.

**Figure 4.**
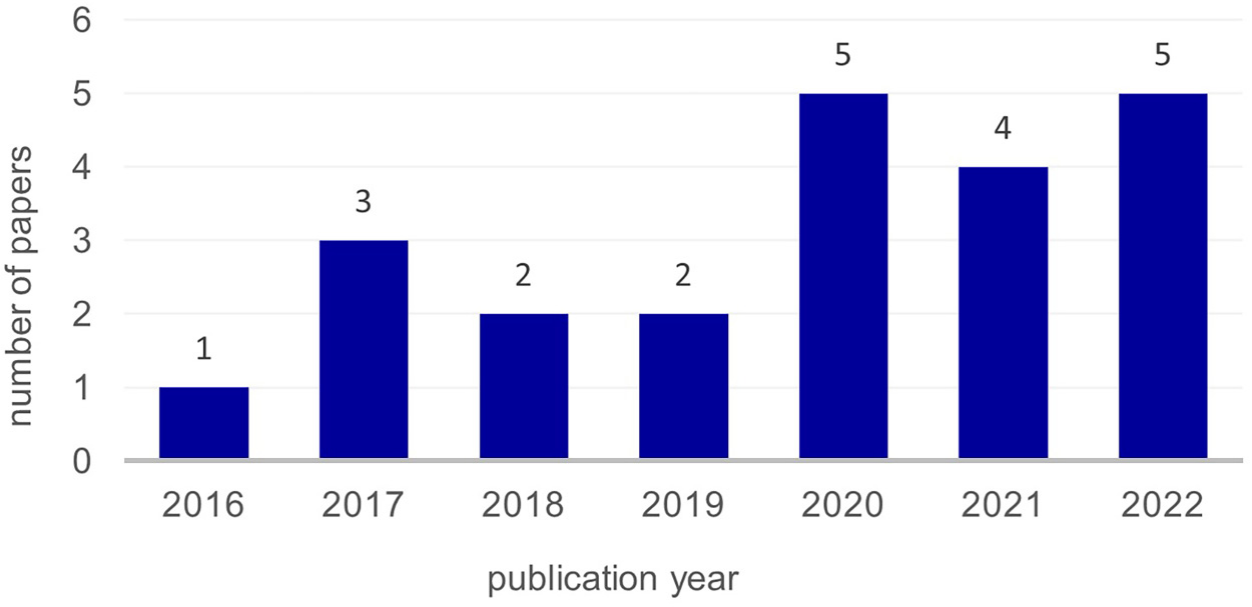
Temporal distribution.

Figure 3 uses a world map to show the nationality of the first author of each of the selected papers. This figure provides information on the geographical/spatial and temporal distribution of the studies addressing VR-based mindfulness interventions on health outcomes. A total of six nationalities were identified, of which North American and South Korean researchers show the largest output concerning VR-based mindfulness interventions. European interest in this field of research has so far been limited to Spanish and French researchers. Figure 4 provides insights on the publication evolution of the selected papers between 2016 and 2022; most of the research was published during the past 3 years (2020–2022; *n* = 9).

The selected papers are distributed through a variety of different publication channels. Among the identified publication sources, *Frontiers in Psychology* is represented three times.^74,76,79^ Another 11 papers were published through different journals like *Pain Medicine*, *Virtual Reality* or *Digital Health* (see Table 3). Four papers were published through the ACM (Association for Computing Machinery) Conference on Human Factors in Computing Systems (CHI) between 2017 and 2022.^78,81,82,85^ Two additional papers were drawn from IEEE (Institute of Electrical and Electronics Engineers) Conferences, one from the Conference on Engineering in Medicine and Biology Society in 2018 and another one from the Conference on Virtual Reality and 3D User Interfaces in 2021.^71,90^ Moreover, two more papers were published through the ACM Symposium on Virtual Reality Software and Technology in 2017 and 2020.^73,75^ Around 64% were published in journals, 27% were presented in conferences and only 9% in symposia. Consequently, journals appear to be the primary source where articles on VR-based mindfulness interventions and their effects on psychological and physiological outcomes are published.

**Table 3.** Publication source of the selected studies.

Publication source	Type	Paper no.	Number	%
ACM Conference on Human Factors in Computing Systems	Conference	^3,9,16,17^	4	18.18
Frontiers in Psychology	Journal	^13,15,20^	3	13.64
ACM Symposium on Virtual Reality Software and Technology	Symposium	^11,18^	2	9.09
IEEE Engineering in Medicine and Biology Society	Conference	^8^	1	4.55
IEEE Virtual Reality and 3D User Interfaces	Conference	^7^	1	4.55
Children	Journal	^21^	1	4.55
Cyberpsychology, Behavior and Social Networking	Journal	^10^	1	4.55
Digital Health	Journal	^22^	1	4.55
Frontiers in Virtual Reality	Journal	^19^	1	4.55
Journal of Military, Veteran and Family Health	Journal	^4^	1	4.55
Pain Medicine	Journal	^2^	1	4.55
Pain Management Nursing	Journal	^6^	1	4.55
Palliative Medicine	Journal	^5^	1	4.55
PLoS One	Journal	^14^	1	4.55
Psychological trauma: theory, research, practice and policy	Journal	^12^	1	4.55
Virtual Reality	Journal	^1^	1	4.55

#### Methodological mapping

##### Research design, sampling and study design

The aim of experimental research is to analyze and understand the effect of a program, treatment or intervention. Three types of experimental research designs are being distinguished in our studies: preexperimental, quasi-experimental and true experimental.^92,93^ In total, 50% preexperimental (pre–post studies with no control group: *n* = 9; case series: *n* = 2), 27% quasi-experimental (*n* = 6) and 23% true-experimental studies (RCTs: *n* = 5) were identified in our selected papers. A methodological gain of quasi- and true-experimental studies can be explained through the addition of a control group. The following section takes a closer look at the sampling process of participants as well as to design specific aspects like sample size, randomization and control group conception (see Table 4) in all selected quasi- and true-experimental studies (*n* = 11).

**Table 4.** Control group design and sampling of quasi- and true experimental studies.

Study	Control group design	Sampling	Measurement time points
Chandrasiri et al. (2019)	Random assignment; active control group (mindfulness audio track)	No inclusion criteria Exclusion criteria e.g., psychiatric disorders or psychological treatment Few demographic characteristics	Pre–post
Groninger et al. (2021)	Random assignment; active control group (guided imagery session)	Inclusion criteria e.g., ≥ 18 years, diagnosed with ACC/AHA stage C or D heart failure Exclusion criteria e.g., familiarity with VR, history of motion sickness/seizures/epilepsy Demographic characteristics	Primary outcome: pre–post–24 hour post secondary outcomes: pre–post
Hawes and Arya (2021)	Random assignment; active control group (VR game)	No inclusion/exclusion criteria Few demographic characteristics	Before/after priming
Kazzi et al. (2018)	Randomized order; passive/active control group (seated rest/audio)	Inclusion criteria: healthy Exclusion criteria: predisposition to motion sickness Few demographic characteristics	Study 1: continuously measurement + at 3 minutes within each condition (rest/audio/VR) Study 2: continuously measurement + at 2 minutes (during stress test) + at 6 minutes (during recovery)
Min et al. (2020)	counterbalanced order; active control group (VR scenario)	No inclusion/exclusion criteria Few demographic characteristics No information on sampling	Pretest, during + right after VR simulation, retrospective (after a day)
Mistry et al. (2020)	8 counterbalanced between group conditions; active control group (guided meditation)	Inclusion criteria: 17–22 years No exclusion criteria Information about ethnicity + regularity/familiarity with meditation practice	After VR-/non-VR meditation
Navarro-Harro et al. (2019)	Random assignment; active control group (mindfulness-based intervention group sessions)	Inclusion criteria e.g., 18–65 years, diagnosis of GAD Exclusion criteria e.g., pregnancy, receiving other psychological treatment Demographic characteristics	Pretreatment–posttreatment MBI + VR group: before/after each session
Roo et al. (2017)	Counterbalanced order; active control group (spatial augmented reality)	Inclusion criteria: interest in meditation No clear exclusion criteria Information about regularity with meditation practice	After VR/SAR session
Tarrant et al. (2018)	Nonrandom assignment; passive control group (resting)	Inclusion criteria: ≥18 years, moderate level of generalized anxiety Exclusion: e.g., history of head injury, seizure activity or major mental health concerns Two independent rounds of sampling (1st pool: intervention group, 2nd pool: control group) Demographic characteristics	Baseline–pre–post
Tarrant et al. (2022)	Alternately assignment; active control group (guided audio meditation)	Inclusion criteria: ≥ 18 years Exclusion criteria e.g., any history of seizure, current blindness or severe eye impairment or history of motion sickness Demographic characteristics	Pre–post
Zambotti et al. (2022)	Counterbalanced order; active control *condition* (quiet activity)	Inclusion criteria: DSM-5 (with/without insomnia) Exclusion criteria: e.g., severe medical condition Demographic characteristics	Before–during–after intervention/control

As part of the sampling process, the application and clear formulation of inclusion and exclusion criteria strongly differ between those 11 selected studies. For example, Hawes and Arya^71^ or Min et al.^73^ do not mention any inclusion or exclusion criteria at all and although Roo et al.^81^ or Mistry et al.^86^ do cite inclusion criteria, exclusion criteria are not discussed further. However, a whole set of clear inclusion and exclusion criteria for participation is present in studies like Groninger et al.,^87^ Kazzi et al.,^90^ Tarrant et al.^76,83^ or Zambotti et al.^88^ as can be seen in Table 4. A similar diverse pattern can be observed in a comprehensive and clear presentation of the demographic characteristics of the study cohort.

**Figure 5.**
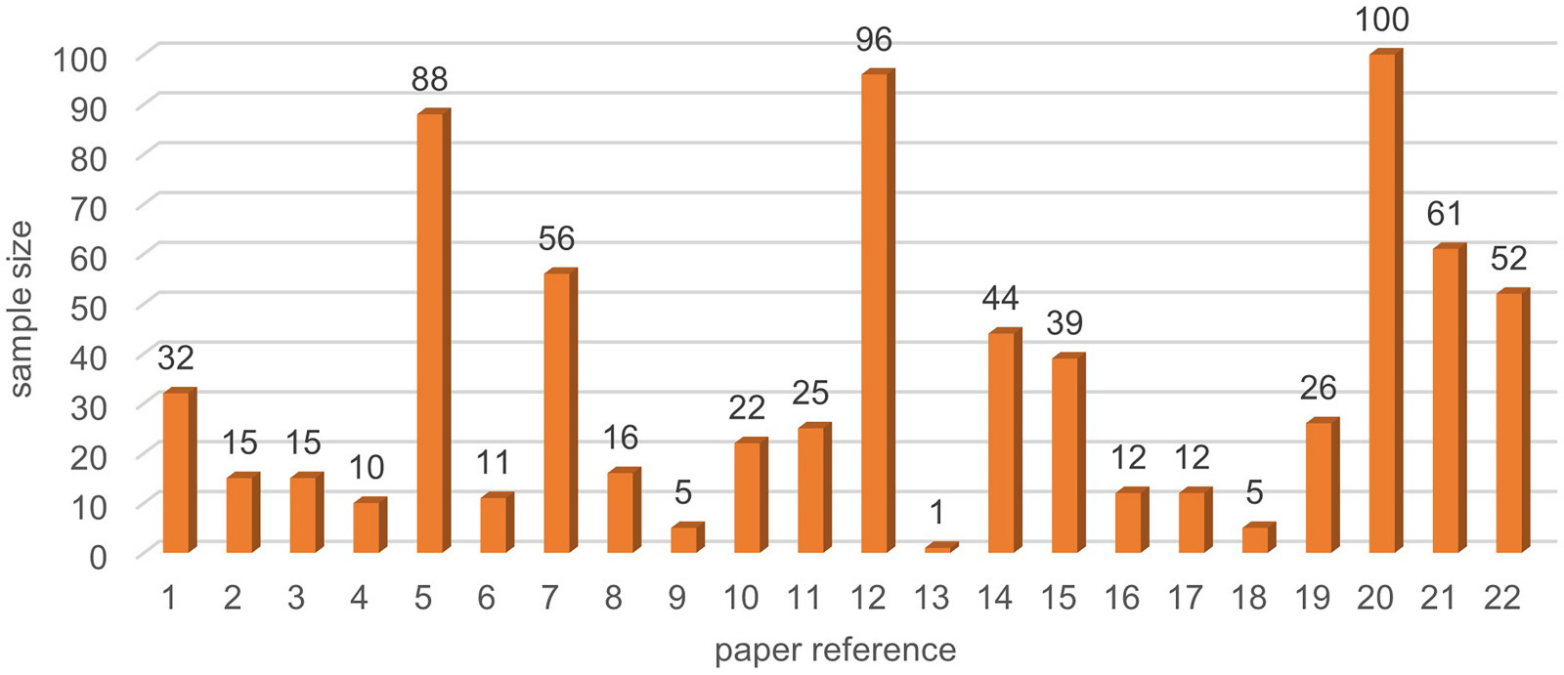
Total sample size of selected studies.

Regarding study design, total sample size varies widely (*M* = 34, SD = ±34, minimum = 1, maximum = 100) in all 22 selected studies (see Figure 5).

Furthermore, the control group design consists of different combinations of active/passive or (non) randomized/counterbalanced/alternating group allocations: (a) active + randomized (*n* = 5), (b) active + counterbalanced (*n* = 4), (c) active + alternating (*n* = 1), and (d) passive + nonrandomized (*n* = 1). Out of 11 quasi- and true-experimental studies, 10 out of 11 studies choose an active control group and so only one study had a passive, resting, control group.^76^ The active control group conditions predominantly comprised audio-based interventions, such as guided meditations.^77,86,87,90^ However, alternative interventions using VR/AR applications,^71,73,81,83^ a quiet activity^88^ or a traditional MBI-intervention^74^ were also administered.

In addition, a closer look was taken at the implementation of multiple measurement time points, which was also a rarely fulfilled criterion in the risk of bias assessment. Broadly, within this systematic review, most quasi- and true-experimental studies primarily measure their variables before and after the intervention, with some additionally measuring physiological variables during the intervention.^73,88,90^ However, the inclusion of baseline measurements as an initial status^76^ and follow-up measurements^73^ is remarkably rare.

##### Subjective vs. objective methods

In our selected studies, 14 papers^69,74,75,77–81,83–87,91^ relied exclusively on subjective data (64%), while seven studies^70–73,76,82,88^ used a combination of subjective and objective data collection methodologies (32%) and a single study^90^ exclusively utilized objective data, representing 4% of the total studies. Subjective data primarily encompass psychological health outcomes such as anxiety, various emotions and mindfulness, while objective data predominantly pertain to the recording of physiological health outcomes, including blood pressure, saliva composition and HRV. A comprehensive outline of all employed subjective instruments and their respective objectives is provided in the Online Supplementary materials, with certain instruments being utilized across multiple studies.

#### Content mapping

##### Outcome variables

Directly linked to the measuring instruments are the respective outcomes representing psychological and physiological health variables. To identify possible outcomes of higher empirical evidence, especially psychological outcomes are classified and assigned to their respective underlying construct, in case only dimensions were measured.

**Figure 6.**
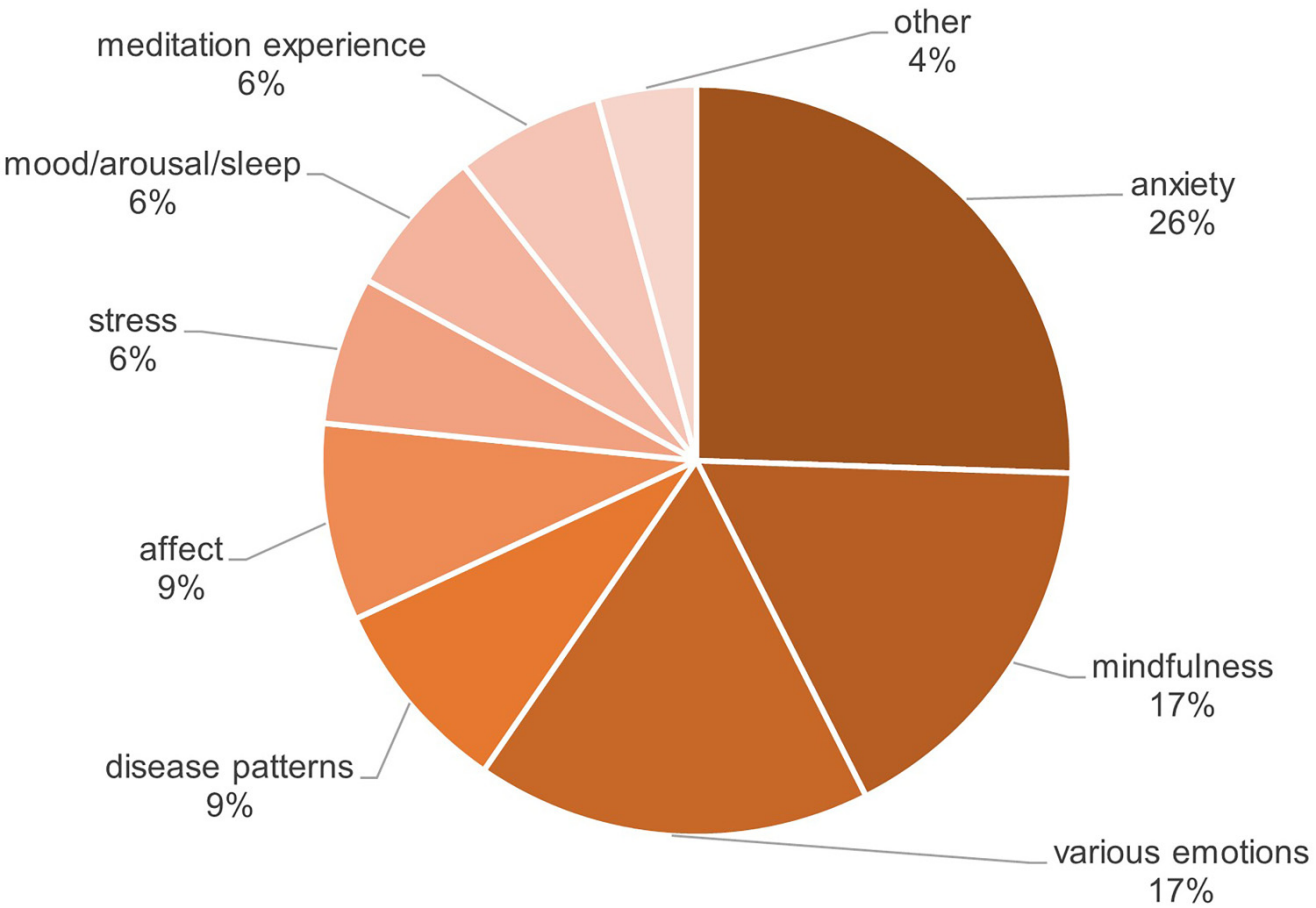
Psychological outcomes.

As shown in Figure 6 anxiety (26%), mindfulness (17%) and various emotions (e.g., anger, surprise or happiness; 17%) are those three types of psychological variables that are addressed the most in the selected papers. Positive effects on anxiety were found in methadone maintenance treatment (MMT) patients,^70^ (non)students,^71^ children,^72^ general anxiety disorder (GAD) diagnosed patients,^74,76^ children and young adults with inflammatory bowel disease^69^ as well as panic disorder patients.^75^ The target group and the setting are strongly clinical, but there is also an application example from a school setting.^72^ Improved mindfulness was found in a general population^77,78,80,81^ as well as in a prediseased population.^74,79^ The positive influence of VR-based mindfulness application on various emotions were mainly found in prediseased participants,^70,74,79^ but also in attendees of an international meeting and frontline healthcare workers.^80,83^ Those three major psychological outcome categories are followed by disease patterns (e.g., depression, posttraumatic stress disorder [PTSD]: 9%), affect (9%), stress (6%), mood/arousal/sleep (6%), meditation experiences (6%) and other (e.g., quality of life, opioid craving; 4%).

**Figure 7.**
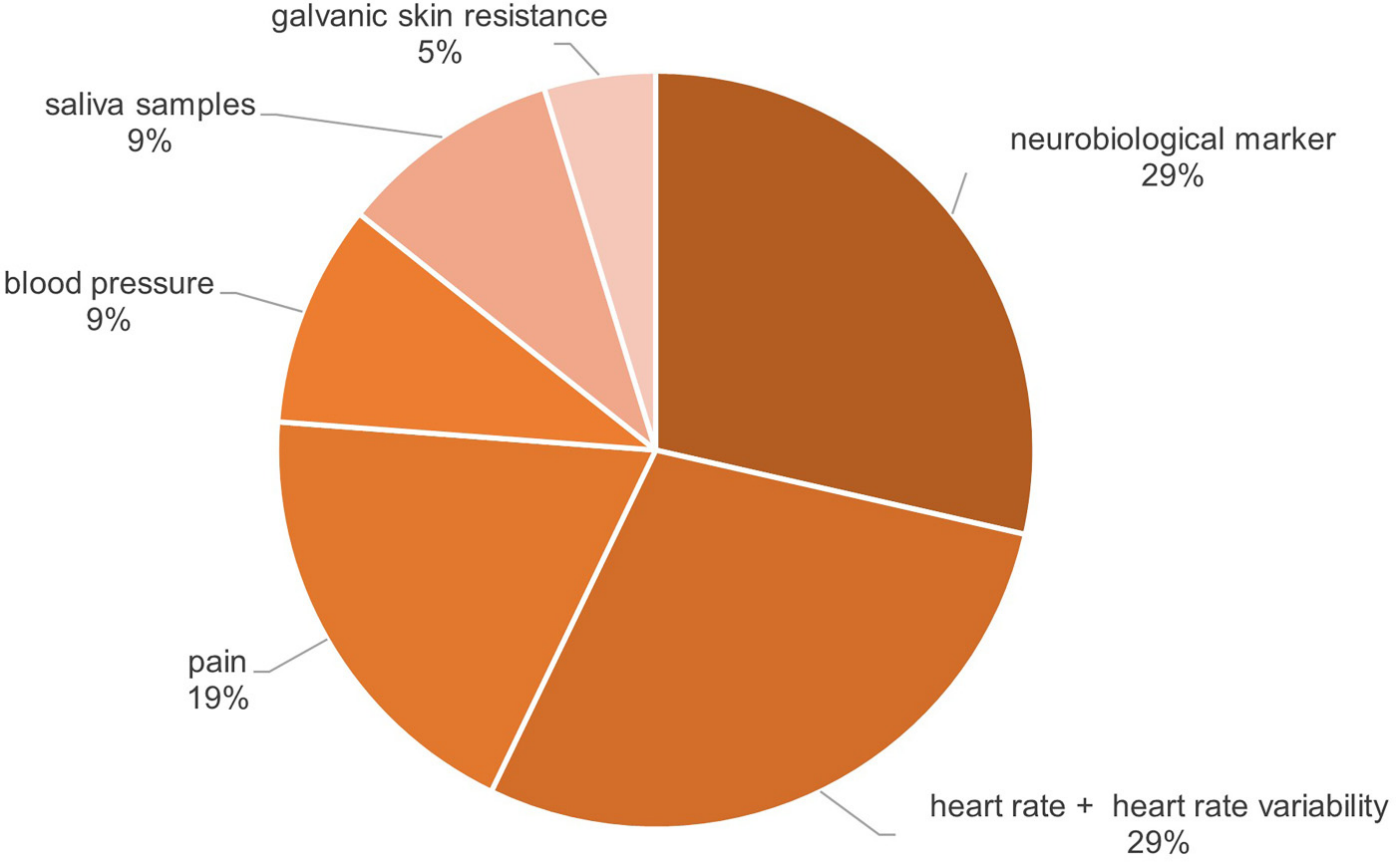
Physiological outcomes.

In the area of physiological health variables (see Figure 7) neurobiological markers (brain-waves, pain neuromatrix activation, resting-state connectivity, cognitive bandwidth; 29%) and heat rate/HRV (29%) were primarily assessed, followed by pain (19%), blood pressure (9%), saliva samples (9%) and galvanic skin resistance (5%). Positive effects on neurobiological markers were found in healthy^71,82,88,90^ and prediseased participants.^70,76,88^ Participants who improved from a VR-based mindfulness application in HR and HRV can be mainly allocated to a healthy collective.^72,73,88,90^

##### VR scenario: content and intensity

Ultimately, the focus will be on VR scenarios from a content-oriented perspective. This will involve an initial analysis of the incorporation of mindfulness within the VR scenarios, followed by an investigation into the structural aspects of the VR protocol concerning its duration and intensity.

Mindfulness is typically cultivated in formal meditation practices like sitting and walking meditation, loving kindness meditation or mindful movements.^3^ Meditation is often used as an umbrella term, under which a number of diverse practices are summarized, but three main criteria have been defined as essential to any meditation practice: (a) the use of defined techniques, (b) logic relaxation meaning not intending to analyze, judge or expect and (c) a self-induced state or mode.^94^ In accordance with the two-component model by Bishop et al.,^5^ Hölzel et al.^95^ identified an array of distinct but interacting mechanisms that are at play in producing the benefits of mindfulness meditation practice. The first mechanism is the attention regulation, seen as a building block for practitioners to also benefit from the other mechanisms of mindfulness practice. It comprises a sustaining attention on the chosen object and whenever attention is distracted, one is able to return attention to the object. The second mechanism, body awareness, includes the focus on an object of internal experience like sensory experiences of breathing, emotions or other body sensations. The third mechanism emotion regulation is divided in the aspect of reappraisal and the aspect of exposure, extinction and reconsolidation. Reappraisal means that one is approaching ongoing emotional reactions in a different way, with acceptance and no judgment. Exposure, extinction and reconsolidation are about exposing oneself to whatever is present in the field of awareness, letting oneself be affected by it and refraining from internal reactivity. The last mechanism, change in perspective on the self, includes the detachment from identification with a static sense of self.^95^ The components might interact very closely with one another, so that a distinction between each component might seem artificial, but at this point we try to identify which components are mostly addressed in the implemented VR scenarios in our selected papers as one of the mechanisms might move into the foreground, while others become less relevant.

**Figure 8.**
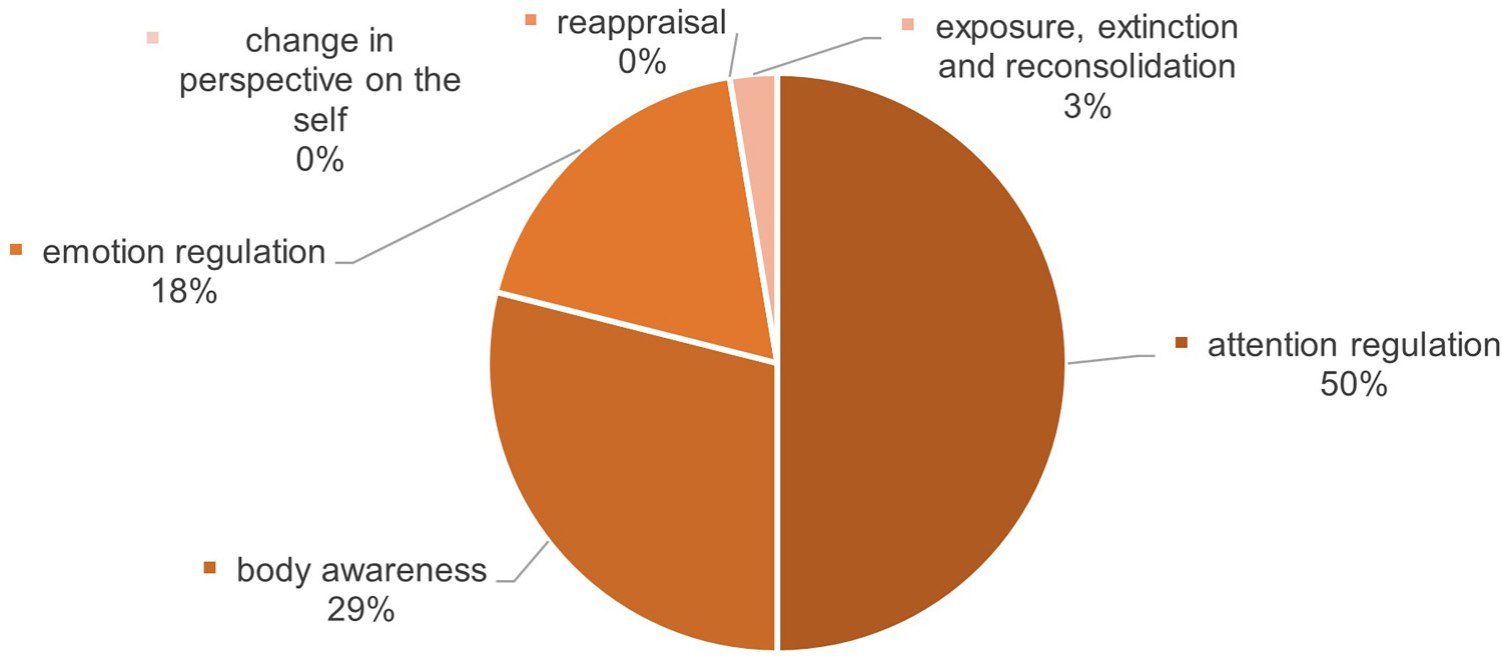
Mindfulness mechanism in virtual reality (VR) scenarios.

A comprehensive list of the VR contents within the included studies, alongside our identified predominant mindfulness mechanisms, is available in the Online Supplementary materials. Among the VR scenarios investigated, attention regulation emerges as the most prevalent mindfulness mechanism, observed in 50% of the included studies, followed by body awareness at 29%, emotion regulation encompassing both facets at 18% and exposure, extinction and reconsolidation at 3%. Notably, mindfulness mechanisms such as “reappraisal” and “change in perspective on the self” were not clearly identifiable in the studies (see Figure 8).

**Figure 9.**
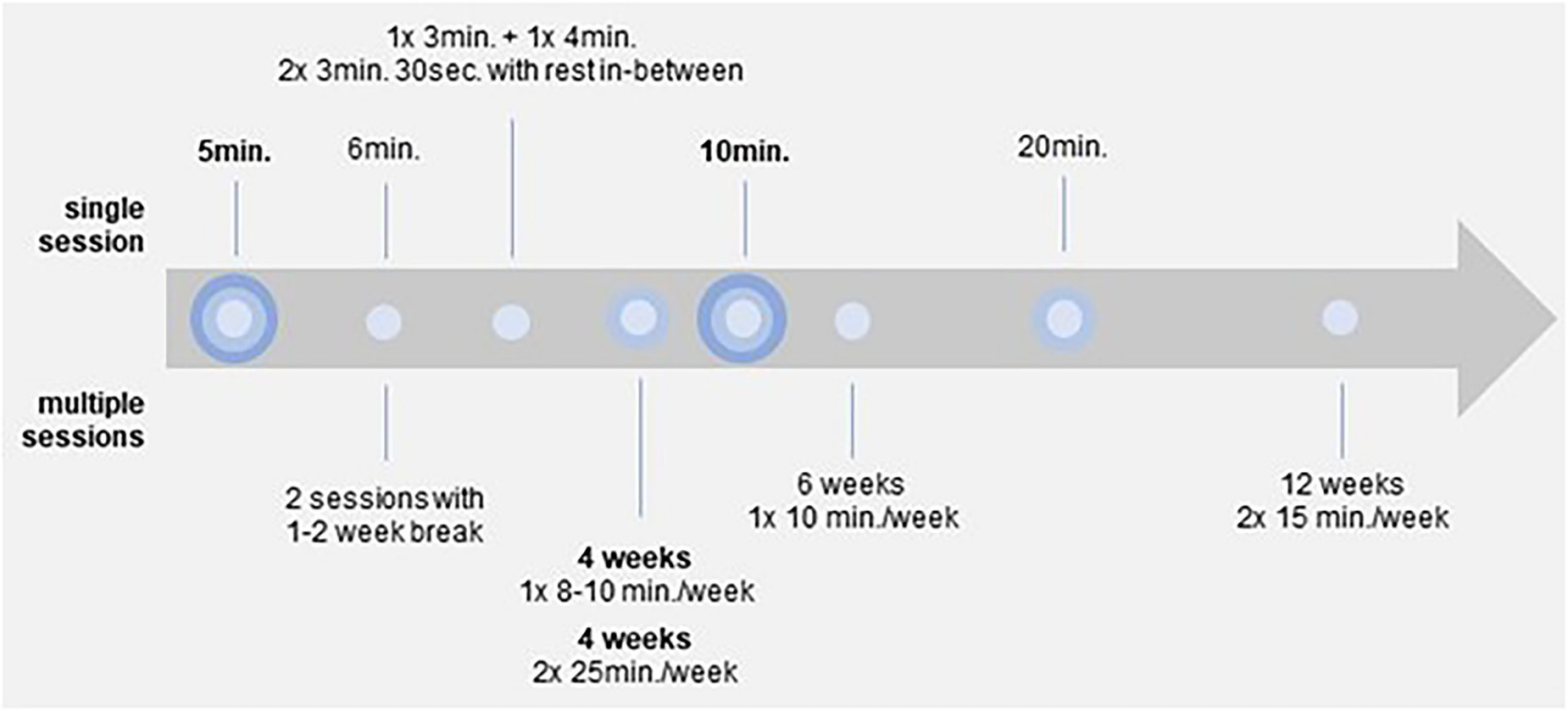
VR session designs.

Regarding the VR protocol, the majority of the selected papers (see Figure 9) conduct only a one-session intervention program (73%) while only a handful run a multiple-session intervention program (27%). The latter is found in the study by Hargett and colleagues,^91^ who include two VR sessions in their study protocol, but do not provide any further details on the specific duration of the individual sessions. In addition, there are no inferential statistical results available, only descriptive data are presented (two sessions with 1–2-week break). In the area of single-session studies, durations of 5^71,76,83,84,86,90^ and 10 minutes^76,80–82,91^ per session are the most common ones. Significant effects on one's health differ from outcomes like pain, emotions/emotional state, mindfulness and presleep arousal in the 5-minute intervention studies. Studies that used a duration of 10 minutes have recorded significant results in health-related variables such as anxiety, HRV, affect/affective states, meditation experience, brain waves and mood.

## Discussion

The following section discusses the results that emerged from the comprehensive evidence mapping of the included studies and highlights the resulting implications for practical application as well as the prospects for further research in the field of VR-based mindfulness interventions and their effects on psychological and physiological health outcomes. It is important to note that a meta-analysis was not performed as the included studies were very heterogeneous in terms of methodological design and contents and therefore could not be compared with each other. Attempting a meta-analysis under these circumstances would likely lead to results with limited power and could potentially mask true effects.

### Distributional mapping of time, space and publication

Although we planned to include studies published from the earliest available, we have only captured those published in the last 6 years (2016–2022). However, immersive reality like HMDs may be seen to have reached a period of high popularity in 2016 and wireless HMDs mainly have been developed in 2017.^96^ The early stage of this technology and its accessibility are reflected in the small number of published research papers, particularly in the area of methodologically rigorous studies. This scarcity clearly underlines the need for additional research efforts in this area.

Regarding regional expertise in VR-based mindfulness interventions and their impact on health variables, this can be particularly found in the North American and South Korean regions judging by the first author's countries of origin. Implementing new technologies, like immersive VR, into all kinds of settings like medicine, education, sports or rehabilitation can be a valuable tool for mental health and physical well-being as their decreasing costs lead to more popularity and accessibility.^90,97^ Expertise and evaluation of such new multidisciplinary tools are warranted, as for example the need for therapy places in the therapeutic area strongly indicates.^98^ Therefore, research groups, especially in the European area, are needed to build on these initial promising results to pave the way for innovative technologies such as VR in the healthcare sector.

According to the findings reported, a valuable insight has been gained as regards to VR-based mindfulness interventions and their effect on psychological and physiological health outcomes, as it was investigated in different publication channels including journals, conferences and symposia. The majority of the papers (64%) selected in this systematic review have already been published in journals focusing on medicine, psychology or virtual/digital topics. Nevertheless, nearly 40% are published through conferences and symposia, which might be explained by the strong preexperimental characteristics around half of the studies have. In addition, among the papers published through conferences and symposia there was only one RCT.^71^ According to our subsequent research, none of these studies were subsequently published in journals.

### Methodological mapping

#### Research design, sampling and study design

With 50% preexperimental studies, 27% quasi-experimental studies and only 23% true-experimental studies, it is imperative to strengthen the basis of empirical evidence by increasing the conduct of quasi- and true-experimental studies.

Within the scope of sampling, both quasi- and true-experimental studies undergo an evaluation of applied inclusion and exclusion criteria. As a full set of clear inclusion and exclusion criteria was not always available in our selected studies, we recommend for future studies to distinctly define their target demographic and precisely articulate the criteria for inclusion and exclusion. Even if this may have happened in the background, transparent sharing of this information is important. This approach aims to facilitate the identification of the study’s target population and enable other researchers to understand the characteristics described that could impact on the study’s objectives. Moreover, insufficiently articulated study descriptions might hinder the ability to faithfully replicate the research and subsequently, practitioners encounter heightened challenges when attempting to effectively implement these methodologies.^99^

Numerous studies in this analysis exhibit a pilot and feasibility nature. According to Hertzog^100^ suggesting sample sizes of 20–25 participants for demonstrating intervention efficacy within a single group and 30–40 participants per group for between-group effect size estimation, such sizes are infrequently observed. Even with these recommended sizes, imprecision in estimates should be acknowledged. Julious^101^ recommends a minimum of 12 participants per group for pilot studies in a parallel group trial. This design is scarcely observed in the selected studies, except for Tarrant et al.^76^ and Mistry et al.^86^ Among studies with single-group designs and pilot or feasibility attributes (*n* = 9; pre–post studies without control groups), only three meet Hertzog’s^100^ threshold.^69,72,80^ However, the goal of feasibility and pilot studies is crucial in critically assessing sample size, as these studies focus on identifying implementation issues and assessing the feasibility of conducting a larger trial. Feasibility studies aim to assess whether an RCT of a particular intervention in a specific setting is viable. Pilot studies replicate the intended final design of the full RCT and serve as a rehearsal to ensure that key components, such as eligibility assessment, randomization procedures and follow-up assessments, function effectively.^100,102^ Even if some studies do not reach certain thresholds in terms of sample size, important findings have been gathered that now need to be deepened in larger trials.

In consideration of additional aspects of study design, the incorporation of a control group should be pursued more in future studies as only half of our studies (*n* = 11) implemented a control group. Using a control group has multiple benefits like enhancing internal and external validity, increasing statistical power and helping to make causal inferences. Moreover, a control group minimizes error variance, strengthens intervention effects and shows what might have happened without the intervention.^103^ However, employing a control group comes with drawbacks that should be taken into account when planning a study. It can raise costs, introduce ethical and practical challenges, create biases, threaten study validity and limit the findings’ applicability. Control groups can assume either an active or passive form. In the context of our research inquiry, which primarily centers on the investigation of VR-based mindfulness interventions, the inclusion of active control groups, sharing identical content with the experimental condition but omitting the VR component, offers valuable insights into the specific efficacy of this technological aspect. Furthermore, the introduction of an additional passive control group, if feasible, would facilitate a more comprehensive exploration of our research question. It is noteworthy that none of the studies under review incorporated this dual-control group approach, although 10 out of the 11 studies did employ active control groups. In three of these studies, the content of the intervention and control conditions remained the same, with the only difference in the type of presentation being that non-VR vs. VR only received an audio-based version.^83,86,87^ This aspect should also be taken into account in the prospective planning of studies.

Randomization is crucial for creating comparable groups and eliminating biases in treatment assignments.^104^ In our selected studies, five used a randomized assignment,^71,74,77,87,90^ four used counterbalancing^73,81,86,88^ and one study each used alternating^83^ and nonrandomized assignment.^76^ Those different randomization techniques all have their certain strengths and it is the researchers’ task to find an appropriate technique for the particular study as was done in most of the included studies with control groups.^103^

Multiple measurement time points play an important role in experimental studies, because they allow researchers to track changes and developments over time. They can also help control confounding variables, identify trends and patterns in data, detect long-term and inter-individual effects and optimize experiments. In addition to the conventional pre–post survey design, a minority of the quasi- and true-experimental studies incorporate supplementary data collection time points, including baseline assessments,^76^ measurements conducted during the intervention^73,90^ and follow-up evaluations.^87^ In forthcoming research, the integration of multiple measurement time points should be considered, particularly in studies characterized by the inclusion of multiple sessions within their design. This approach enhances the capacity to effectively evaluate temporal changes over the course of the study.

#### Subjective versus objective methods

The focus of the health outcomes examined in the included studies was clearly in the psychological area, consequently a substantial prevalence of subjective measurement instruments was observed in these studies. Given the inherent complexity of psychological constructs, they often elude direct observation, rendering their measurement a nontrivial task. Although subjective measurement methods have some limitations or perceived disadvantages like their limited interpretation due to their ordinal scale, they are nevertheless indispensable in many cases to capture personal experiences, attitudes and opinions.^105^ The critical view of subjective measurements often refers to the need to carefully check their validity and reliability and possibly to complement them with objective measurements in order to obtain a more comprehensive picture.^106^ In our case, the concurrent utilization of both subjective and objective methodologies for the assessment of psychological and physiological variables has the potential to enhance the comprehensive understanding of an individual’s health status. Nevertheless, the validity of subjective measures can be independent of their relationship to objective measures. Consequently, subjective measures can serve the empirical purposes they were meant to serve.^105^ Most of the subjective measures that were applied in the selected studies are evaluated and standardized tools like Mindfulness Awareness Attention Scale (MAAS),^107^ Beck Anxiety Inventory (BAI)^108^ or Generalizied Anxiety Disorder Scale-7 (GAD-7)^109^ with good psychometric properties.

Furthermore, we would like to discuss the concepts of “subjective and objective measures” as researchers often use those terms to distinguish between procedures, that either rely on human judgment or record data with a diagnostic instrument. Since there is no clear consensus on what is an “objective” and what is a “subjective” result, there is rather a continuum between the two terms. It is clear from this that subjectivity and objectivity are not definitively delineated binary distinctions, which leads us to introduce the conceptual terms “captured by human” and “captured by device” for consideration.^110^ In conclusion, we would like to state that we share the view, that the types of variables to be assessed in any given study should be determined on the basis of the hypotheses being tested, not on opinions about the value of different data collection strategies.^111^

### Content mapping

#### Outcome variables

Anxiety seems to be the psychological health outcome with the highest empirical evidence^69–76^ in our included studies, notwithstanding the need to acknowledge that, to some extent, distinct facets or dimensions of anxiety were assessed. This fact must also be taken into account for all other variables. Nevertheless, in 18% of our studies the reduction of anxiety through VR-based mindfulness interventions was demonstrated with large effect sizes (*d* > |.96|/*η*_p_^2 ^= .60). With regard to the validity of these effects, one study used a true-experimental design (RCT)^74^ and two a quasi-experimental design^73,76^ and only one study^72^ was carried out with a preexperimental design. A further four studies addressed anxiety in their study,^69–71,75^ which would make a total of 36% of all studies that addressed anxiety as a health variable. The majority of these additional studies utilized preexperimental designs without control groups. Despite their ability to identify significant changes, they did not provide information on effect sizes. Thus, there is at least some robust evidence for the internal validity of the anxiety-reducing effect of VR-based mindfulness interventions that of course needs to be replicated in future methodologically rigorous studies.

Medium to large effects on mindfulness or related constructs (*d* > .75), such as interoceptive awareness, were found in 14% of the studies. This statement is based on two preexperimental studies^78,80^ and one true-experimental study^74^ (RCT). In two additional studies where mindfulness was considered a psychological health variable (constituting 23% of the total), no effect sizes were disclosed, despite the fact that these studies were either quasi-experimental^81^ or true-experimental studies^77^ and identified significant effects. At this stage, the evidence of the internal validity of a mindfulness-promoting effect of VR-based mindfulness interventions still needs to be expanded.

A similar picture of results can be seen for the variable various emotions/emotional state, as the positive effects of VR-based mindfulness interventions could be shown by 18% of our studies with medium to large effects (*d* > .51). Regarding validity of these effects, two studies used a true-experimental design^74,83^ and two were carried out with preexperimental design.^80,82^ Two further studies,^79,84^ which would account for a total of 27% of all studies, dealt with emotions in their study, but only at a descriptive level. Consequently, there exists substantial evidence supporting the internal validity of the favorable impact of VR-based mindfulness interventions on emotions or emotional states. It is essential, however, that this evidence be substantiated through methodologically rigorous studies in the future.

For disease patterns, in particular depression, one true-experimental study^74^ (RCT; 5%) reported a medium to large effect (*d* = |.54|). This suggests a promising internal validity of the results, but also needs to be strengthened by further methodologically rigorous studies and extended to other disease patterns. Depression was documented by three additional preexperimental studies^70,72,75^ (constituting 18% of the total), but without any indication of effect sizes due to partial lack of inferential statistical evaluation. Besides depression, PTSD symptoms also fall into this category, but were reported in only one study on a descriptive level.^84^

In 9% of our studies the positive effect of VR-based mindfulness interventions on affect was demonstrated with large effect sizes (*d* = .90|/*η*^2 ^> .338). With regard to validity of these effects, one study used a quasi-experimental design^86^ and one study used a preexperimental design.^82^ Consequently, the evidence for the internal validity of this positive effect on affect needs more foundation. Nevertheless, affect was addressed in two further studies,^84,85^ but only evaluated on a descriptive level.

In each case, 5% of the studies showed a positive effect on stress^78^ respectively presleep arousal^82^ with medium to large (*d* = .55) respectively small to medium (*d* = .28/*d* = .35) effects. Both were preexperimental studies, which does not directly support the internal validity of the effects and therefore more methodologically rigorous studies in the future are needed. Significant effects on distress could be found in another study,^87^ but was not supported by effect sizes. The absence of effect sizes was similarly observed in another preexperimental study,^84^ which exclusively provided descriptive values for stress variables. Regarding presleep arousal, another quasi-experimental^88^ study that incorporated this variable into the study design failed to reveal any additional evidence, as no significant effects were observed.

In 9% of our studies VR-based mindfulness interventions support the ability to meditate respectively a positive meditation experience with large effect sizes (*d* > 1.43/*η*^2 ^> .323). Validity of these effects is based on one study carried out with a quasi-experimental^86^ and one with a preexperimental^78^ study design. In order to strengthen the internal validity of these effects, more methodologically rigorous studies should be pursued.

In our last category, other, no effect sizes are mentioned for variables such as quality of life or opioid craving. However, the latter is a very specific variable that only affects a certain target group and not as universally applicable as, for example, mindfulness or emotions.

As already mentioned in some instances statistically significant effects were discerned within the purview of the investigated psychological health outcomes, however no effect sizes could support them. Eleven studies alone did not provide any information on this, four of them exclusively reported their findings at a descriptive level, without engaging in statistical analysis.^75,79,84,85^ This entails only limited inference, difficulties in replication and only limited possibilities for generalization. Statistical analyses are required to increase the significance and reliability of the results. They make it possible to test hypotheses, identify patterns and distinguish statistically significant results from random fluctuations. While descriptive results can provide important initial information, they should be considered only as a first step in the analysis of research data. In addition, a complete reproduction of information in the statistical analysis is important for the classification of the results and should be aimed for in future studies.

Although the range of physiological variables is comparatively less diverse, they rarely denote a superordinate aspect of health or a superordinate construct. For this reason, the scope for interpretation of physiological health outcomes is diverse and is not further defined in many articles—in most cases they stand for themselves. For example, neurobiological markers can provide information about neurological health status, while HRV, salivary metrics, galvanic skin resistance, heart rate and blood pressure can serve as potential markers of (acute) stress and relaxation.^112^ In addition, EEG spectra can also provide information about relaxation and stress.^113^ For this reason, we recommend an even stronger theoretical embedding of the variables for future studies.

Nevertheless, in 9% of our studies the improvement of EEG-data based variables through VR-based mindfulness interventions was demonstrated with large effect sizes (*η*_p_^2 ^> .14). With regard to the validity of these effects, those two studies were carried out with a quasi-experimental study design.^76,88^ More large effects in the category of neurobiological markers could be detected in correlation-based relationships within pain neuromatrix activations (*r* > .557) based on a preexperimental study design.^70^ Although partially significant results on cognitive bandwidth were found in a true-experimental study^71^ (RCT), no information on effect sizes is available. In another preexperimental study,^82^ EEG data were recorded but only documented at a descriptive level. Taking all these studies together, a total of 23% of all studies deal with neurobiological markers, but not all of them report or perform a complete inferential statistical analysis. Thus, there is only some slight evidence for the internal validity of the positive effect of VR-based mindfulness interventions on neurobiological markers that of course needs to be deepened in future methodologically rigorous studies.

In one quasi-experimental study^88^ and therefore in 5% of our included studies, large effects in heart rate (*η*_p_^2 ^= .19) and medium to large effects in HRV (*η*_p_^2 ^> .10) were reported. Even if two further quasi-experimental studies^73,90^ and one preexperimental study^72^ show partially significant results in these two variables, these are accompanied by no further information on the effect size. Consequently, the internal validity of these effects has not yet been sufficiently confirmed and requires further methodologically rigorous studies.

Four studies, constituting 18% of all studies, included pain as a health variable in their design, including three preexperimental^69,70,91^ and one true-experimental studies.^87^ Nevertheless, only one preexperimental study^70^ (5%) reported a large effect size (*η*^2 ^= .265), although significant effects were found across all four studies. This has not yet yielded strong evidence supporting the internal validity of the pain-reducing impact of VR-based mindfulness interventions, emphasizing the need for further research in the future.

Positive effects of VR-based mindfulness interventions on cortisol could be shown with large effects (*η*^2 ^= .62/*η*_p_^2^ = .25) by 9% of our studies. With regard to the validity of these effects, one study used a quasi-experimental design^88^ and one study was only carried out with a preexperimental design.^70^ Thus, there is only slight evidence for the internal validity of the cortisol-reducing effect of VR-based mindfulness interventions that of course needs to be replicated in future methodologically rigorous studies.

Although inferential statistical analyses were partially successful in identifying significant changes in physiological health variables such as blood pressure and galvanic skin resistance within three quasi-experimental studies,^73,88,90^ they were not supported by effect sizes. To enhance the internal validity of the effects of VR-based mindfulness interventions on those variables, upcoming studies should furnish comprehensive statistical documentation.

#### VR scenarios: content and intensity

Even though attention regulation was present in half of all VR scenarios and is considered a basic mechanism for all other mechanisms, an appropriate VR scenario should always be tailored to the study objective.^46^ Depending on the target group, e.g., beginners/advanced learners or healthy/prediseased people, VR contents can be less or more suitable. Equally important is the desired mechanism in order to be able to induce targeted effects and make them measurable.

Conspicuous across most VR scenarios is the nature aspect that significantly defines the virtual environment. Nature can be characterized as “areas containing elements of living systems that include plants and nonhuman animals across a range of scales and degrees of human management, from a small urban park through to relatively ‘pristine wilderness.’”^114^ The possible interaction between mindfulness and exposure to nature might be explained by the psychological mechanism “attention” which is suggested to underlie positive effects of both mindfulness training as component of trait mindfulness and exposure to nature as part of attention restoration.^115–117^ These findings currently exist superficially with tangible real nature, but they manifest the potential for a meaningful transfer to VR as evidenced by van Rompay et al.^118^ Building on the attention restoration theory^119^ and stress recovery theory,^120^ both central to people–environment research, these approaches highlight the importance of vastness and spaciousness for the beneficial effects of nature interaction on mental health and well-being. van Rompay et al.^118^ demonstrated that VR simulations of spacious landscapes can enhance selflessness and related measures such as connectedness and positive affect. This mediation effect of spaciousness on selflessness and positive affect is explained by an embodied process in wherein body boundaries loosen up.^121^ This process is associated with awe, a “nonbasic emotion”^122^ respectively a sense of being in the presence of something greater than oneself and integrating elements of self-transcendence.^123^ Considering this in the context of mindfulness mechanisms, particularly the change in perspective on the self, which was difficult to identify directly in our VR scenarios, some parallels can be drawn. Spacious VR-based nature scenarios may help address the static sense of self by promoting reduced self-centeredness and a heightened sense of connectedness with the broader world.^124,125^ Experiencing nature-based awe and achieving a state of self-transcendence may lead individuals to adopt a more dynamic sense of self. This shift, initiated by spacious nature-based VR scenarios, may not only enhance mindfulness but also offer various other mental health benefits. This approach is supported by Chirico and Gaggioli’s^126^ assumption that VR can facilitate so-called transformative experiences (TEs). These are defined as brief, extraordinary and unique events that lead to durable and/or irreversible outcomes, contributing to changes in an individual’s self-conception, worldviews and perspectives on others as well as their personality and identity. TEs involve an expansion of knowledge about oneself, others and the world (epistemic expansion) and are characterized by heightened emotional complexity, which includes emotional variability, high intensity and mixed emotions as their core phenomenological features.^127^ These experiences emerge through the combination and manipulation of specific emotional and epistemic affordances. Notably, emotional affordances that elicit complex emotions such as awe are more readily implemented in VR settings than in real-world environments. This perspective is embedded in Gaggioli et al.’s transformative experience design^128^ model, from which it can be deduced, as outlined above, that the mechanism of mindfulness, the change of perspective on the self, can be addressed through a VR-based awe experience. In addition to changes in perspective on the self, another mechanism that can benefit from these experiences is reappraisal, which was also challenging to identify directly in our studies. This mechanism involves adopting an accepting and nonjudgmental approach to ongoing emotions. The spectrum of transformative effects is comprehensive, manifesting on both emotional and behavioral levels. This can positively influence an accepting and nonjudgmental approach to emotions, while also highlighting the complexity involved in designing and structuring virtual realities.^126^ The challenge in identifying these two mindfulness mechanisms may stem from our focus on predominantly recognizable and identifiable mechanisms, potentially leading to the prioritization of more accessible and fundamental mechanisms such as attention regulation or body awareness. Moreover, obtaining clear identification information, that could be relevant for change of perspective on the self and reappraisal is difficult without personal insight into the scenarios or detailed descriptions of the virtual environment and content. However, this should be considered by future developers of VR-based mindfulness scenarios who aim to incorporate these specific mechanisms. Consequently, critical questioning, including the integration of desired emotional and epistemic affordances or nature-based awe, should be incorporated into the development process. For instance, to evoke a sense of awe, the virtual environment should be designed to convey a sense of vastness and promote cognitive accommodation, thereby helping users transcend familiar schemas of the world, themselves and others.^126^ Additionally, emotional VR scenarios viewed from a first-person perspective (experiencer) rather than a third-person perspective (observer) have the potential to enhance mental involvement and influence behavioral intentions.^129^ Although explicit information on the user’s perspective was not given in our included studies, it can be assumed that in most cases it is a first-person perspective. To sum it up, current studies seem to apply VR-based mindfulness scenarios with focus on especially three out of six possible mindfulness mechanisms, namely attention regulation (50%), body awareness (29%) and emotion regulation (18%). However, as attention regulation is seen as a building block for practitioners to benefit from the other mechanisms of mindfulness practice,^95^ it is only natural and important to integrate this mechanism into the VR scenarios in order to appeal to a wide range of users.

In addition to natural scenarios, specific environments could also find their way into VR in the future and make the content even more target group-specific addressing the aspect of personalized interventions.^130^

Many of our conducted studies have primarily employed single-session methodologies, demonstrating the immediate beneficial effects of brief VR-based mindfulness interventions. For future research, a focused exploration on short-term mindfulness interventions is recommended, incorporating for example a self-regulated training protocol enabling participants to engage in repeated and consistent VR-based mindfulness practices over an extended duration rather than a singular session. Adopting such an approach would facilitate the examination of the effects of VR-based mindfulness interventions on psychological well-being and especially the immediate (state) and enduring (trait) mindfulness. The work of Cahn and Polich,^131^ for example, supports the idea that sustained and comprehensive meditation training contributes to a lasting improvement in cognitive abilities and general well-being. In addition to further investigation of possible short-term effects on health outcomes, various long-term multi-session protocols should also be conducted, as the empirical basis in this area requires more evidence. The overall goal of future studies is to identify an appropriate dose–response relation for different target groups.

### Study limitations

As with any review, this study entails several considerations that could potentially constrain the robustness of the conclusions drawn. In addition, this review is meant not as a complete description of the research in the field but to stimulate scientific debate, especially on a methodological level.

With regard to our primary aim of providing a comprehensive insight into this specific area of research, the main bias affecting the study’s conclusions arises from the incompleteness of the search process and the selection of studies. Despite conducting the trial search across major digital libraries pertinent to health psychology and human–computer interaction themes, a possibility of overlooking relevant studies remains. Furthermore, achieving complete exhaustiveness in the search was unattainable, even though the PICO criteria were employed to formulate a comprehensive search string aimed at gathering a diverse array of relevant elements.

Moreover, potential bias during the data extraction phase might have influenced the accuracy of the extracted data. To mitigate this concern, two authors (AW and FS) engaged in discussions to establish consensus on the data items for extraction in this study. However, as the data extraction was conducted by a single reviewer, a risk of errors that could introduce considerable inconsistencies in the outcomes exists. Single data extraction processes can potentially lead to extraction errors, such as inadequacies, incomplete data and omissions. While it’s common for systematic reviews to employ duplicate data extraction by at least two reviewers independently, following PRISMA guidelines, it’s noteworthy that some reviews in other areas have utilized single data extraction with subsequent double checking, yielding significant results.^132,133^ The use of single data extraction presents a practical approach to complete reviews within constraints of time and cost when implementing a double-extraction process may not be feasible. Possible bias can also be noticed in conclusions regarding the presence or absence of relationships. To address this concern, the study has carefully managed any phase where there could be disagreement between the authors, ensuring discussions until a consensus was reached. Additionally, apart from textual descriptions, the use of various charts to represent results aimed to strengthen the connection between extracted data and the study’s conclusions, enhancing traceability.

From a content point of view, the possible side effects of VR should also be briefly discussed at this point. In addition to the numerous positive effects described, VR also always harbors risks or adverse effects, which must be taken into account in terms of effectiveness and safety. As summarized by Simón-Vicente et al.^134^ in their review article, VR side effects are more likely to be observed with HMDs than with desktop systems. For this reason, we screened all articles for possible adverse effects, looking in particular for symptoms from the Simulator Sickness Questionnaire^135^ such as discomfort, fatigue, headache, eyestrain, dizziness or nausea.

In eight of our 21 included studies adverse effects were reported. They reach from discomfort of the headset,^77^ hypervigilance, mildly elevated feelings of embarrassment and self-consciousness in the presence of the assessor while wearing the HMD to sadness, anger, irritation, guilt and nervousness in PTSD patients.^84^ In Hawes and Arya’s^71^ study, eight out of 56 participants felt some level of discomfort and one participant experienced some cyber nausea and had to stop the experiment. Moreover, symptoms like motion sickness and headache were also present, but only to a small extent. Within the study of Kim et al.,^85^ where a VR mindfulness game with mysterious stones was used, some participants reported distracting elements such as visuals. Adverse side effects, such as distraction, were documented in the study by Mistry et al.,^86^ affecting only 22.9% of participants. Similar findings were reported by Semertzidis et al.,^82^ where distracting bodily sensations were associated with the inter dream system. In this system, participants interact with graphic images projected in space and VR through EEG manipulation while resting in an interactive bed. Other side effects were documented by Wren et al.,^69^ with a single participant reporting symptoms such as dizziness and increased anxiety following the VR sessions. Three studies^87,90,91^ incorporated potential adverse effects within their exclusion criteria. Consequently, individuals predisposed to such side effects or those with prior negative experiences with VR were systematically excluded from participation. One study explicitly mentioned that there were no adverse effects from the use of VR.^75^

On a positive note, even when side effects did occur, they were usually very mild and did not prevent the participants from completing the experiment, with the exception of one person. Nevertheless, it is important to take precautions to ensure the safe and enjoyable use of VR and to reduce the likelihood of undesirable effects. The specific target population plays a decisive role in this context, just as it does in the selection of a suitable VR content.

Besides those adverse effects, the VR scenarios within our included studies all have inherent mindfulness aspects as shown by our content analysis. From a technological perspective, an important question is how these VR scenarios can support the cultivation of mindfulness practice. While mindfulness is superficially characterized by internal aspects such as interoceptive perception and decentering, it also includes external components like focused attention. Conversely, VR and its environment are primarily associated with external aspects. Those challenges and opportunities have been reviewed by Döllinger et al.^136^ showing that VR provides promising characteristics that can support mindfulness and related health outcomes. Particularly, HMDs offer advantages in shielding external extractors.^137^ For example, visual cues can make it possible to subtly direct the user’s focus, which is more effective than audio-only meditation instructions or visual guidance on screen. In addition, bodily and mental states can be promoted by adding neuro-/biofeedback. However, embodying a virtual avatar or lacking any visual body reference might distract the user from their physical body and self-awareness/focus.^138^ So, the created VR/AR/MR experience should be carefully designed to maintain focus and avoid creating new distractions with overly complex elements. In their systematic review, Döllinger et al.^136^ explain a comprehensive range of influencing factors in the development of such scenarios, which leads to a kind of design guideline. These factors include types of virtual environments, objects, self-representation and the presence of other people as well as guidance, feedback and interactivity. In addition, they highlight other important influences, such as individual user characteristics, including previous meditation experiences and the synthesis of effects. Given the abundance of these factors, this is not part of this systematic review, but it should be considered, especially for future developments of VR-based mindfulness interventions.

### Implications of the results

The results of this systematic literature review, which includes a comprehensive mapping and classification analysis, are relevant for researchers and practitioners in the field of VR-based mindfulness interventions to enhance psychological and physiological well-being.

As the temporal and geographic mapping has shown, this field of research is still in its infancy, which is why the initial but important findings of our studies should be deepened and expanded. From a methodological perspective, researchers need to pay considerable attention on their experimental design strategy including aspects such as sample size, sampling, a transparent reporting of inclusion and exclusion criteria, the incorporation of active and passive control groups and the implementation of multiple measurements and follow-up assessments. The selection of appropriate measurement tools also constitutes a critical methodological consideration. It is essential to acknowledge that simultaneous employment of both subjective and objective methods for evaluating psychological and physiological variables holds promise for enriching the comprehensive understanding of an individual’s health status. Furthermore, from a content perspective, future research should focus on additional studies involving short- and long-term intervention protocols to provide more precise dosage recommendations.

For a brief theoretical classification of VR-based mindfulness interventions in the health context, we would like to refer again to Antonovsky’s salutogenesis model^28^ mentioned at the beginning, as a corresponding model under the aspect of digitalization, in particular with the inclusion of immersive VR, is currently still pending. Our research underscores the potential for VR-based mindfulness interventions to function as GRRs, supporting movement toward the health ease pole of the so-called health ease/dis-ease (HEDE) continuum by strengthening the SOC, despite SOC not being directly measured in our included studies. Mindfulness induced through VR scenarios assumes a mediating role by enhancing the regulation of psychological health outcomes such as emotions, fears, affect and stress. This improved regulation and adaptive response fosters more effective coping mechanisms, thereby contributing viewing life as more manageable. By cultivating present-moment attention and awareness, mindfulness can help individuals to better comprehend and respond to challenging experiences like illness or pain. Moreover, these beneficial effects extend beyond pain management to include physiological parameters like HRV and blood pressure, improving overall health improvements across the continuum. The mediating influence of mindfulness, observed in traditional settings, is similarly evident in the digital respectively virtual world, whereby the psychological effects may be more accessible or better observable for humans as for example a reduced blood pressure or increased HRV might not so easily perceived by everyone. An example highlighting mindfulness's capacity to foster psychological adaptations and enhance coherence can be found in the study by Weissbecker et al.,^139^ which examined its effects among women with fibromyalgia.

Regardless of practitioners’ specific interest in particular variables, target groups or VR content, this systematic review provides a good opportunity to create relevant filters and filter out studies that meet their specific needs. It enables practitioners to identify and select studies that are most suitable for their intended applications and objectives. In addition, they have the task to create and validate diverse virtual environments, each tailored to accommodate target-specific elements or incorporating biofeedback mechanisms within the scenario. This integration enables guided practices to dynamically respond to users’ physiological states, such as adjusting to the user’s breathing patterns, thereby enhancing the immersion experienced by the user within the virtual environment.^140^ Furthermore, these findings should be passed on and translated into other settings.

Compared to both existing reviews, that are quite close to our research question, there is an overlap of nine papers with Arpaia et al.^45^ (53 in total) and six papers with Zhang et al.^44^ (nine in total). A total of 13 out of 22 of our studies cannot be found in either of them. This deviation is partly due to the temporal dimension of the publication date of these studies, which goes beyond the time frame of the two aforementioned reviews. Nine studies from 2021 and 2022^69–71,78,83,85,87,88,91^ alone cannot be included in the two reviews. This confirms the assertion made at the beginning that this field of research is developing dynamically and rapidly. In terms of content, Zhang et al.'s^44^ review primarily identified psychological health effects on depression, stress, emotions or anxiety. Physiological health effects, on the other hand, were limited to EEG data (*n* = 1), respiratory rate (*n* = 1) and pain levels (*n* = 1). Especially new findings on physiological variables such as galvanic skin response, salivary cortisol, blood pressure or heart rate and HRV could be identified in the data we included. Findings from the narrative review by Arpaia and colleagues focus on the research question of how VR technology can improve mindfulness practice respectively therapeutic effects of mindfulness, particularly those of interoceptive awareness and decentering. Furthermore, technological design solutions in VR-based mindfulness training will be discussed. With regard to these research questions, physiological and psychological health outcomes are mentioned, but the focus lies on the therapeutic effect/clinical target group and the technological implementation, from which further design proposals are then derived. Clinical use cases include chronic/acute pain, anxiety, stress, depression, borderline and addiction. Even if Arpaia et al.'s^45^ work has a different focus, the group of authors also comes to the conclusion that this field of research has the need for more rigorous, randomized controlled studies in the future.

## Conclusion

This paper reports on a systematic literature review that summarizes the existing research regarding VR-based mindfulness interventions and their effects on psychological and physiological health outcomes. From an initial set of 949 papers retrieved from six databases, 22 studies were selected. These papers were investigated using a hermeneutic approach for conducting literature reviews including evidence mapping with distributional, methodological and content analyses.

Regarding our studies, it has been observed that since 2016, researchers, particularly in the North American and South Korean regions, have shown increasing interest in VR-based mindfulness interventions and their impact on psychological and physiological health outcomes. The majority of related publications have been published through journals, with around half of them adopting a preexperimental research design. There is an obvious need for further empirical evidence to support and extend the existing findings. Future studies should take into account important methodological parameters such as sample size, sampling, inclusion and exclusion criteria, control groups, randomization or multiple measurement times. This aspect also includes the selection of appropriate measurement instruments, which in our studies were mostly of a subjective nature. Simultaneous use of subjective (human-captured) and objective (device-captured) methods can help to obtain a holistic picture of the state of health and should be taken into account in future studies.

Studies show a higher level of evidence in the area of psychological health outcomes, particularly for anxiety, mindfulness and various emotions. These findings are mainly supported by methodologically more rigorous studies that fully document the results of their statistical analyses, including the reporting of effect sizes (see paper numbers 15, 19 and 20). However, even in true-experimental studies (RCTs like paper numbers 1, 5 and 7), this full range of information is not always available and makes it challenging to classify the results. The majority of the positive effects in terms of physiological health variables can be seen in neurobiological markers and heart rate or HRV. Two papers (numbers 2 and 22) in particular make an important contribution to this by fully reflecting health effects at a statistical level, although these are not true-experimental study designs.

In the VR scenarios, the mindfulness mechanism of attention regulation was superficially represented, while the environment was strongly characterized by natural elements. VR protocols with single sessions and durations of 5 and 10 minutes were the most common, but both short- and long-term programs should be further investigated in future studies in order to make more specific dose–response statements.

In summary, the included studies have provided important initial findings and show that this young field of research requires further studies to secure and expand the evidence base.

## Supplemental Material

sj-docx-1-dhj-10.1177_20552076241272604 - Supplemental material for Psychological and physiological health outcomes of virtual reality-based mindfulness interventions: A systematic review and evidence mapping of empirical studies Supplemental material, sj-docx-1-dhj-10.1177_20552076241272604 for Psychological and physiological health outcomes of virtual reality-based mindfulness interventions: A systematic review and evidence mapping of empirical studies by Alissa Wieczorek, Florian Schrank, Karl-Heinz Renner and Matthias Wagner in DIGITAL HEALTH
