# Supplementary material for: Psychological and physiological health outcomes of virtual reality-based mindfulness interventions: A systematic review and evidence mapping of empirical studies
Source: Digit Health. 2024 Oct 25;10:20552076241272604. doi: 10.1177/20552076241272604 (PMC11526413; doi:10.1177/20552076241272604)
Supplement: sj-docx-1-dhj-10.1177_20552076241272604 - Supplemental material for Psychological and physiological health outcomes of virtual reality-based mindfulness interventions: A systematic review and evidence mapping of empirical studies [file sj-docx-1-dhj-10.1177_20552076241272604.docx]

***Quality Assessment Tool for Before-After (Pre-Post) Studies with no control group***

|  | Faraj et al., 2021 | Feinberg et al., 2022 | Hargett et al., 2022 | Kim et al., 2022 | Kwon et al., 2020 | Navarro-Haro et al., 2017 | Semertzidis et al., 2019 | Seol et al., 2017 | Wren et al., 2021 |
| --- | --- | --- | --- | --- | --- | --- | --- | --- | --- |
| 1. study question | yes | yes | yes | yes | yes | yes | yes | yes | yes |
| 2. eligibility criteria and study population | yes | yes | yes | no | yes | yes | yes | yes | yes |
| 3. study participants representative of clinical populations of interest | yes | yes | no | no | yes | yes | CD | yes | yes |
| 4. all eligible participants enrolled | yes | yes | yes | CD | yes | yes | yes | yes | no |
| 5. sample size | no | yes | no | no | no | yes | yes | no | no |
| 6. intervention clearly described | yes | yes | yes | yes | yes | yes | yes | yes | yes |
| 7. outcome measures clearly described, valid, and reliable | yes | yes | yes | yes | yes | yes | yes | yes | yes |
| 8. blinding of outcome assessors | no | no | no | no | no | no | no | no | no |
| 9. follow-up rate | NA | yes | NA | NA | NA | NA | NA | NA | NA |
| 10. statistical analysis | yes | yes | yes | no | yes | yes | yes | no | yes |
| 11. multiple outcome measures | yes | yes | no | no | yes | no | no | no | no |
| 12. group-level interventions and individual-level outcome efforts | NA | NA | NA | NA | NA | NA | NA | NA | NA |
| **Quality Rating (Rater 1 + Rater 2)** | **good** | **good** | **fair** | **poor** | **good** | **good** | **good** | **poor** | **fair** |

***Quality Assessment for Case Series Studies / JBI critical appraisal tool***

|  | Frewen et al., 2020 | Narraro-Haro et al., 2016 |
| --- | --- | --- |
| 1. Were there clear criteria for inclusion in the case series? | unclear | unclear |
| 2. Was the condition measured in a standard, reliable way for all participants included in the case series? | yes | yes |
| 3. Were valid methods used for identification of the condition for all participants included in the case series? | yes | yes |
| 4. Did the case series have consecutive inclusion of participants? | unclear | no |
| 5. Did the case series have complete inclusion of participants? | unclear | yes |
| 6. Was there clear reporting of the demographics of the participants in the study? | no | yes |
| 7. Was there clear reporting of clinical information of the participants? | no | yes |
| 8. Were the outcomes or follow up results of cases clearly reported? | yes | yes |
| 9. Was there clear reporting of the presenting site(s)/clinic(s) demographic information? | no | yes |
| 10. Was statistical analysis appropriate? | unclear | unclear |
| **Quality Rating (Rater 1 + Rater 2)** | **exclude** | **include** |

***Quality Assessment for Quasi-Experimental Studies / JBI critical appraisal tool***

|  | Kazzi et al., 2018 | Min et al., 2020 | Mistry et al., 2020 | Roo et al., 2017 | Tarrant et al., 2018 | Zambotti et al., 2022 |
| --- | --- | --- | --- | --- | --- | --- |
| 1. Is it clear in the study what is the ‘cause’ and what is the ‘effect’? | yes | yes | yes | yes | yes | yes |
| 2. Were the participants included in any comparisons similar? | yes | yes | yes | unclear | yes | unclear |
| 3. Were the participants included in any comparisons receiving similar treatment/care, other than the exposure or intervention of interest? | yes | yes | yes | yes | yes | yes |
| 4. Was there a control group? | no | no | no | no | yes | no |
| 5. Were there multiple measurements of the outcome both pre and post the intervention/exposure? | no | no | no | no | yes | no |
| 6. Was follow up complete and if not, were differences between groups in terms of their follow up adequately described and analyzed? | NA | yes | NA | NA | NA | NA |
| 7. Were the outcomes of participants included in any comparisons measured in the same way? | yes | yes | yes | yes | yes | yes |
| 8. Were outcomes measured in a reliable way? | yes | yes | yes | yes | yes | yes |
| 9. Was appropriate statistical analysis used? | yes | yes | yes | yes | yes | yes |
| **Quality Rating (Rater 1 + Rater 2)** | **include** | **include** | **include** | **seek further info** | **include** | **seek further info** |

***Quality Assessment for Randomized Controlled Trials / JBI critical appraisal tool***

|  | Chandrasiri et al., 2019 | Groninger et al. 2021 | Hawes & Arya, 2021 | Navarro-Haro et al., 2019 | Tarrant et al., 2022 |
| --- | --- | --- | --- | --- | --- |
| 1. IV: Was true randomization used for assignment of participants to treatment groups? | yes | yes | yes | yes | no |
| 2. IV: Was allocation to treatment groups concealed? | unclear | yes | unclear | unclear | unclear |
| 3. IV: Were treatment groups similar at the baseline? | unclear | yes | unclear | yes | yes |
| 4. IV: Were participants blind to treatment assignment? | unclear | no | unclear | unclear | unclear |
| 5. IV: Were those delivering the treatment blind to treatment assignment? | unclear | unclear | unclear | unclear | unclear |
| 6. IV: Were treatment groups treated identically other than the intervention of interest? | yes | yes | yes | yes | yes |
| 7. IV: Were outcome assessors blind to treatment assignment? | unclear | yes | unclear | yes/  unclear | unclear |
| 8. IV: Were outcomes measured in the same way for treatment groups? | yes | yes | yes | yes | yes |
| 9. IV: Were outcomes measured in a reliable way? | yes | yes/ unclear | yes | yes/  unclear | yes |
| 10. IV: Was follow up complete and if not, were differences between groups in terms of their follow up adequately described and analyzed? | NA | yes (pain) | NA | yes (emotions) | NA |
| 11. CV: Were participants analyzed in the groups to which they were randomized? | yes | yes (pain) | yes | yes | yes |
| 12. CV: Was appropriate statistical analysis used? | yes | yes | yes | yes | yes |
| 13. CV: Was the trial design appropriate and any deviations from the standard RCT design accounted for in the conduct and analysis of the trial? | yes | yes | yes | yes | yes |
| **Quality Rating (Rater 1 + Rater 2)** | **include** | **include** | **include** | **include** | **include** |

IV = Internal Validity; CV = Statistical Conclusion Validity

***Applied Subjective Measures***

| **Subjective Measures** | **Aim** |
| --- | --- |
| **BAI** = Beck Anxiety Inventory | measuring clinical anxiety |
| **BASS** = Buddhist affective states scale | measuring affective states described within Buddhist psychology that were not included in the mDES |
| **BRUMS** = Brunel Mood Scale | 32 mood descriptors that are categorized into 8 unipolar dimensions: anger, tension, depression, vigor, fatigue, confusion, happiness and calmness |
| **CAMS-R** = Cognitive and Affective Mindfulness Scale-Revised | measuring a broad conceptualization of mindfulness with language that is not specific to any particular type of meditation training |
| **(adapted) DBT® diary card** | measuring BPD-related (borderline personality disorder) urges and primary emotions |
| **DERS** = Difficulties of Emotion Regulation Scale | 36-items scale measuring emotion dysregulation; five subscales: inattention, confusion, non-acceptance, interference, impulse |
| **DISS** = Daytime Insomnia Symptom Scale | measuring state alert cognition/negative mood/positive mood/sleepiness + fatigue (VAS) |
| **Emotional State Scale**  (1-7 points VAS) | how they felt at that moment for the following emotions: happiness, sadness, anger, surprise, anxiety, relax/calm vigor/energy |
| **FACIT-Pal14** = Functional Assessment of in Chronic Illness Therapy in Palliative Care 14 item scale | measuring general quality of life in palliative care patients |
| **FFMQ** = Five Facets of Mindfulness Questionnaire | multifactorial scale measuring five facts of mindfulness: observe, describe, act with awareness, non-judging of inner experience, and non-reactivity to inner experience |
| **GAD-7** = General Anxiety Disorder 7 Items | measuring the severity of generalized anxiety disorder |
| **K-MADRS** = Korean Version of the Montgomery-Asberg Depression Scale | measuring the severity of depressive episodes |
| **HADS** = Hospital Anxiety and Depression Scale | measuring symptoms of anxiety and depression |
| **HAM-A** = Hamilton Anxiety Rating Scale | measuring the severity of anxiety symptoms |
| **KIMS** = Kentucky Inventory of Mindfulness | 39-item measuring mindfulness on four scales: observing, describing, act with awareness and accept without judgment |
| **MAAS** = Mindfulness Awareness Attention Scale | measuring characteristic of dispositional mindfulness, namely, open or receptive awareness of and attention to what is taking place in the present |
| **MAIA** = Multidimensional Assessment of Interoceptive Awareness | 32-items multidimensional assessment of interoceptive awareness with eight factors: noticing, distracting, non-worrying, attention, emotional, self-regulation, body-listening, trusting |
| **mDES** = modified differential emotional | measuring extent to which positive and negative emotions have been experienced within a particular time frame |
| **MEQ** = meditative experience questionnaire | measuring normative, everyday kinds of meditative experiences |
| **Meditation questionnaire**  (10-point Likert scale) | measuring meditation ability, overall meditation ability, confidence meditating solo |
| **National Comprehensive Cancer**  **Network Distress Thermometer** | brief screening tool for cancer patients to assess distress |
| **PANAS(-X)** = positive and negative affect schedule (extended) | measuring positive and negative affects |
| **PCL-5** = self-report of symptoms of PTSD | PTSD diagnosis/checklist using the new DSM-5 psychiatric manual |
| **PSAS** = Pre-Sleep Arousal Scale | 16-item instrument measuring cognitive and somatic arousal as a state |
| **PSS** = Perceived Stress Scale | stress assessment instrument |
| **SCL-10R** = Symptom Checklist 10-Revised | measuring psychological distress |
| **Self-reported pain score**  (11-point Likert scale) | one-dimensional assessment of pain intensity in chronic pain, cancer pain and acute pain populations |
| **STAI** = sate-trait anxiety inventory | measuring sate and trait anxiety |
| **TMS** = Toronto Mindfulness Scale | measuring mindfulness with two factors: decentering + curiosity |
| **VAS** = Visual Analogue Scales | pain / opioid craving / anxiety / anger / depression |

***Virtual Reality Content and its Mindfulness Mechanism***

| **No.** | **Reference** | **VR-scenario** | **VR-content** | **mindfulness mechanism** |
| --- | --- | --- | --- | --- |
| **1** | **Chandrasiri et al., 2020** | a walk on the beach | 360° landscape relaxation video of Australia’s coastal; instruction: passively observe the video of beach scenes | attention regulation |
| **2** | **Faraj et al., 2021** | guided meditation | using narration and VR the intervention coordinates physical (arms, hands, upper body) movements with a specific breathing technique (Breath Brake®), and meditative exercises (which are neither distracting nor dissociative) to vividly imagine and act as a powerful martial arts “warrior” who must face down his/her internal personal struggles 🡪 KKC program (Kids kick cancer) | body awareness + exposure, extinction, reconsolidation |
| **3** | **Feinberg et al., 2022** | Zen VR Interaction | VR curriculum: a series of lessons designed to teach to beginners with the basics of posture, breath and attention | attention regulation + body awareness |
| **4** | **Frewen et al., 2020** | Guided meditation VR | guided meditation with self-chosen environment/ music (e.g., tropical forest, hidden cave, island, underwater coral reef, foreign planet) | attention regulation |
| **5** | **Groninger et al., 2021** | Forest of Serenity | guide through a forest and waterfall with voice narration | attention regulation |
| **6** | **Hargett et al., 2022** | Calm® | guided meditation in different environments (mountain lake, redwood forest, white sand beach) including topics like gratitude, impermanence, choice, grief, creativity, letting go, resilience, overcoming adversity + philosophic practices of wabi sabi (Japanese philosophic practice with focuses on the beauty of imperfections and impermanence, encouraging a holistic and transient view of life) and ubuntu (African philosophic practice wherein the individual is viewed as part of a larger and more significant relational, communal, societal, environmental and spiritual world) | attention regulation + emotion regulation |
| **7** | **Hawes & Arya, 2021** | Calm® | participant enters the meditation space on a lofty  mountaintop above a large body of water, they are met with beautiful nature sounds (waves, wind, and birds chirping) + guided meditation (very simple, focusing primarily on noticing the beautiful scenery and bringing attention to the breathing process) | attention regulation + body awareness |
| **8** | **Kazzi et al., 2018** | VR guided relaxation and breathing | Guided relaxation and breathing + calming, animated computer-rendered, artificial virtual environment with vivid pastel colors + female British voice-over artist with a calming an accessible tone | attention regulation |
| **9** | **Kim et al., 2022** | Melody of the Mysterious Stones | VR mini games: 1. finding the exit when exploring a cave by using spatialized sounds as a guide, 2. finding mysterious stones by using spatialized sounds as a hint, 3. tearing down ugly stones with annoying sounds, once completed the player gets a meditation as reward | attention regulation |
| **10** | **Kwon et al., 2020** | VR meditation | guided meditation in exam environment (no further details) | attention regulation + body awareness + emotion regulation |
| **11** | **Min et al, 2020** | Drop the beat | user holds virtually a heart in his hand and directly witness its pounding (haptic device gives the simulated kinesthetic feedback of a beating heart); aural and textual instructions helping to regulate one’s breathing and calm down; actual heart rate is sensed and displayed; goal: one’s heart is not about to explode as feared | attention regulation + body awareness + emotion regulation |
| **12** | **Mistry et al., 2020** | Guided Meditation VR | guided meditation with self-chosen environment/ music (e.g., tropical forest, hidden cave, island, underwater coral reef, foreign planet) | attention regulation |
| **13** | **Narraro-Haro et al., 2016** | VR floating scenario | floating down a 3-D computer-generated river while listening to one of three DBT® mindfulness training audio tracks: 1. observing and noticing sounds + bringing attention back to sounds every time the mind wanders off, 2. observing visuals – watching the thins along the way and learning to bring attention back, if it comes distracted, 3. wise mind “stone flake on the lake” – person is instructed to imagine the he/she is a stone, who is floating down an imaginary lake, which represents the inner wise mind | attention regulation |
| **14** | **Navarro-Haro et al., 2017** | VR floating scenario | floating down a 3-D computer-generated river while listening to one of three DBT® mindfulness training audio tracks: 1. observing sounds, 2. observing visuals, 3. wise mind “stone flake on the lake” (as synthesis of opposites – emotion mind and reasonable mind) | attention regulation |
| **15** | **Navarro-Haro et al., 2019** | VR floating scenario | floating down a 3-D computer-generated river while listening to one of three DBT® mindfulness training audio tracks: 1. observing sounds, 2. observing visuals, 3. wise mind “stone flake on the lake” (as synthesis of opposites – emotion mind and reasonable mind) | attention regulation |
| **16** | **Roo et al., 2017** | Inner Garden | wandering through self-made virtual garden | attention regulation |
| **17** | **Semertzidis et al., 2019** | Inter-Dream system | EEG-activity is represented through VR headset: higher levels of activity = larger/more non-uniformed shaper, higher contrast and greater amplitude of the oscillating movement of the imagery (neurofeedback; artistic representation of EEF activity) | body awareness |
| **18** | **Seol et al., 2017** | Drop the beat | user holds virtually a heart in his hand and directly witness its pounding (haptic device gives the simulated kinesthetic feedback of a beating heart); aural and textual instructions helping to regulate one’s breathing and calm down; actual heart rate is sensed and displayed; goal: one’s heart is not about to explode as feared (upstream peaceful state and panic exposure via VR) | attention regulation + body awareness + emotion regulation |
| **19** | **Tarrant et al., 2018** | VR nature experience | 360° video photography with mountains and large rocks all around on the landscape, blue sky speckled with clouds and mist rolling in front of the mountains + soft piano/violin music + mindfulness meditation with a woman’s voice directing the attention to elements of the environment and asking to connect with what they see by imagining that they embody the same qualities as the rocks and sky | attention regulation + body awareness |
| **20** | **Tarrant et al., 2022** | Healium VR beach relaxation | body-scan/relaxation mindfulness meditation with 360° video photography: beach with rock outcropping on either side with waves gently rolling in, a woman’s voice directing attention to specific parts of the body + inviting relaxation into each part | body awareness |
| **21** | **Wren et al., 2021** | MediMindfulness-Transitions | guided mindfulness practice focused on bringing participants’ attention and awareness to their breath, physical sensations in their body and natural environments (e.g. waterfall in meadow, northern lights) | attention regulation + body awareness |
| **22** | **Zambotti et al., 2022** | Nature Treks VR | guided meditation with paced breathing and different environments | attention regulation + body awareness |
